# DigiLoCS: A leap forward in predictive organ-on-chip simulations

**DOI:** 10.1371/journal.pone.0314083

**Published:** 2025-01-09

**Authors:** Manoja Rajalakshmi Aravindakshan, Chittaranjan Mandal, Alex Pothen, Stephan Schaller, Christian Maass

**Affiliations:** 1 Department of Computer Science and Engineering, Indian Institute of Technology Kharagpur, Kharagpur, West Bengal, India; 2 Department of Computer Science, Purdue University, West Lafayette, Indiana, United States; 3 ESQlabs Gmbh, Saterland, Germany; 4 MPSlabs, ESQlabs Gmbh, Saterland, Germany; Università Campus Bio-Medico di Roma, ITALY

## Abstract

Digital twins, driven by data and mathematical modelling, have emerged as powerful tools for simulating complex biological systems. In this work, we focus on modelling the clearance on a liver-on-chip as a digital twin that closely mimics the clearance functionality of the human liver. Our approach involves the creation of a compartmental physiological model of the liver using ordinary differential equations (ODEs) to estimate pharmacokinetic (PK) parameters related to on-chip liver clearance. The objectives of this study were twofold: first, to predict human clearance values, and second, to propose a framework for bridging the gap between *in vitro* findings and their clinical relevance. The methodology integrated quantitative Organ-on-Chip (OoC) and cell-based assay analyses of drug depletion kinetics and is further enhanced by incorporating an OoC-digital twin model to simulate drug depletion kinetics in humans. The *in vitro* liver clearance for 32 drugs was predicted using a digital-twin model of the liver-on-chip and *in vitro* to *in vivo* extrapolation (IVIVE) was assessed using time series PK data. Three ODEs in the model define the drug concentrations in media, interstitium and intracellular compartments based on biological, hardware, and physicochemical information. A key issue in determining liver clearance appears to be the insufficient drug concentration within the intracellular compartment. The digital twin establishes a connection between the hardware chip structure and an advanced mapping of the underlying biology, specifically focusing on the intracellular compartment. Our modelling offers the following benefits: *i*) better prediction of intrinsic liver clearance of drugs compared to the conventional model and *ii*)explainability of behaviour based on physiological parameters. Finally, we illustrate the clinical significance of this approach by applying the findings to humans, utilising propranolol as a proof-of-concept example. This study stands out as the biggest cross-organ-on-chip platform investigation to date, systematically analysing and predicting human clearance values using data obtained from various *in vitro* liver-on-chip systems. Accurate prediction of *in* *vivo* clearance from *in* *vitro* data is important as inadequate understanding of the clearance of a compound can lead to unexpected and undesirable outcomes in clinical trials, ranging from underdosing to toxicity. Physiologically based pharmacokinetic (PBPK) model estimation of liver clearance is explored. The aim is to develop digital twins capable of determining better predictions of clinical outcomes, ultimately reducing the time, cost, and patient burden associated with drug development. Various hepatic *in vitro* systems are compared and their effectiveness for predicting human clearance is investigated. The developed tool, DigiLoCs, focuses explicitly on accurately describing complex biological processes within liver-chip systems. ODE-constrained optimisation is applied to estimate the clearance of compounds. DigiLoCs enable differentiation between active biological processes (metabolism) and passive processes (permeability and partitioning) by incorporating detailed information on compound-specific characteristics and hardware-specific data. These findings signify a significant stride towards more accurate and efficient drug development methodologies.

## 1 Introduction

The drug testing dilemma presents a significant challenge in pharmaceutical development, marked by high costs and a distressing attrition rate in accurately predicting human responses [1, 2]. A pivotal element in preclinical drug development is the accurate estimation of the first-in-human dose and different dosing regimens to keep drug levels within a therapeutic range. This demands precise assessments of hepatic clearance of the drug and human pharmacokinetics [3, 4]. Typically, the gold standard in drug development is the use of simpler *in vitro* systems to study drug metabolism, including liver microsomes [5] and suspension or plated hepatocytes [6]. The drug depletion data (time-concentration profile) are then analysed to determine the *in vitro* drug clearance rate. A simple mathematical model has been employed in earlier work, and it considers the *in vitro* system as a single compartment, the one-compartment PK model [7]. Well-mixing and instantaneous drug distribution is assumed and all biological processes, e.g., permeability and partitioning from cell culture media into intracellular milieu are lumped into drug clearance. This approach also cannot differentiate between compounds actually being metabolised and compounds bound to media proteins or hardware. The so determined *in vitro* clearance value is then extrapolated to humans (*in vitro*—*in vivo* extrapolation) and integrated into human physiologically-based pharmacokinetic (PBPK) models [4, 8] to predict human pharmacokinetics (absorption, distribution, metabolism and excretion (ADME)), before actually testing a new compound in humans. Although this approach is well-established in drug development and easy to use, it also systematically underpredicts human PK [9] by 5–10 fold across studies and compounds.

Microphysiological systems (MPS) and organ-on-chips, as well as 3D organoids, hold great promise to address more complex *in vitro* ADME, toxicology and pharmacology questions offering miniature, biomimetic systems that replicate key aspects of human organ physiology [10–12]. These technologies create an environment where human cells can grow and interact in an organ-specific context, providing insights into human biology and disease that were previously unattainable in conventional *in vitro* models or animal studies. OoC and MPS-based systems are not only used in today’s drug development for PK but also for assessing drug efficacy and toxicity [10, 13–15]. While the emulated *in vitro* biology of MPS and OoCs is getting ever more complex and produces more human-relevant data, these systems still fall short in considerably improving the prediction power of in-human situations, like PK [13, 16]. However, MPS and OoC data are also still analysed using the conventional mathematical model (one-compartment) that does not account for advanced biology. It remains unclear whether the OoC and MPS biology is still not human-relevant enough (and thus not producing human-relevant data) or the conventional mathematical analysis is the cause for the underprediction. Potentially, a digital twin framework that enables the mapping of complex on-chip biology to advanced mathematical models could provide a useful approach to enable OoC and MPS translation to humans and increase the prediction power, but it is currently lacking.

The current study aims to develop a digital twin approach integrating MPS and OoC data within advanced computational models of biology to improve the prediction of clinical clearances. DigiLoCs, our developed digital liver-on-chip simulator, facilitates the accurate description of on-chip complex biology to disentangle biological processes, namely clearance, permeability, and partitioning. The tool comprises and utilises information on complex biological processes (clearance, permeability, partitioning), hardware-specific information from the studied *in vitro* system, and compound-specific information. By accounting for more multi-dimensional information, the tool enables differentiation between active biological processes, such as metabolism, and passive ones, such as permeability and partitioning of a compound from cell culture media into the cellular environment. This tool offers a significant advancement over conventional approaches, which fail to explicitly consider passive biological processes and conflate them into a singular clearance mechanism. By providing a more detailed understanding of biological processes, our tool has the potential to reveal better insights into liver-chip biology. Drug depletion kinetics of 32 compounds were taken from literature covering commercially available liver-on-chips (CnBio [13, 17], Javelin), and 3D spheroids [18, 19], including fast and slow-cleared compounds. According to these studies, DigiLoCs outperform the conventional prediction approach considerably. The impact of a more accurate description of clinical clearance values on predicting human PK was investigated in a proof-of-concept study using propranolol. The kinetics of propranolol was predicted in humans using the conventional approach, DigiLoCs, and literature approach. The results obtained from DigiLoCs for propranolol in the proof-of-concept study were much closer to the actual observed human values than those of other approaches.

To the best knowledge of the authors, this is the first and biggest study so far, comparing head-to-head the performance of different hepatic *in vitro* systems to predict human clearance and demonstrating the impact OoC and MPS systems can have in the drug development process, enhanced through the modelling and prediction features of DigiLoCs.

## 2 Methods

In this section, the following are described: *i*) data used in the study for predicting human clearance, *ii*) DigiLocs, digital twin for liver-on-chip, *iii*) mathematical model, parameter estimation and sensitivity analysis for DigiLocs, and *iv*) translation to humans and prediction of human pharmacokinetics.

### 2.1 Data

In this work, published data on pharmacokinetics (metabolism) or toxicology studies of 32 drugs are used (See Table 1) to predict human pharmacokinetics.

**Table 1:** Overview of literature reports providing on-chip pharmacokinetic information on compound clearance.

Study	Drugs	Used in this study	*In vitro* system	cell number [a.u.]	Media volume [ml]	Flow	On-chip compartments
Docci et al. 2022 [13]	9	9	CnBio Liver-Chip	3E5	1.60	Recirculation	1
Tsamandouras et al. 2017 [17]	6	3	CnBio Liver-Chip	3E5	1.60	Recirculation	1
Rajan et al. 2023 [20]	12	8	Javelin Liver-Chip	2.15E5	1.30	Recirculation	2
Kanebratt et al. 2021 [18]	4	4	3D Spheroid (Hurel)	6E3	0.05	No	1
Bonn et al. 2016 [19]	8	8	3D Spheroid (Hurel)	3E4	0.10	No	1
Total	38	32					

### 2.2 Mathematical model

A typical single-compartment model is described as follows. Let *C*(*t*) be the drug concentration in the chip at time *t*, *V* the volume of the chip, and CL_*c*_ the clearance parameter. Then we have

$$\begin{array}{c} \frac{dC}{dt}=-\frac{{\text{CL}}_{c}}{V}\cdot C, \end{array}$$

with initial value *C*(*t* = 0) = *C*_0_. The solution to this ODE is

$$\begin{array}{c} C(t)=C_{0}\cdot e^{-\frac{{\text{CL}}_{c}}{V}\cdot t}. \end{array}$$ (1)

Taking the logarithm on both sides,

$$\begin{array}{c} \text{log}\;C(t)=-\frac{{\text{CL}}_{c}}{V}\cdot t+\text{log}\;C_{0}, \end{array}$$ (2)

which is equivalent to regression on log-transformed kinetic data.

A digital twin of liver-on-chip with three compartments that incorporate much more information on parameters related to both on-chip characteristics and drug-specific properties was developed. The three-compartment model considering media, interstitium and intracellular compartment is described as follows: Let *C*_*m*_(*t*), *C*_*i*_(*t*), *C*_*c*_(*t*) and *V*_*m*_, *V*_*i*_, *V*_*c*_ be the concentration of the drug at time *t*, and volume of the media, interstitium, and intracellular compartment respectively. CL_*c*_ is the clearance parameter. Then we have

$$\begin{array}{cc} \frac{dC_{m}}{dt} & =-\frac{k_{1}}{V_{m}}\cdot C_{m}+\frac{k_{2}}{V_{m}}\cdot C_{i}; \end{array}$$ (3)

$$\begin{array}{cc} \frac{dC_{i}}{dt} & =\frac{k_{1}}{V_{i}}\cdot C_{m}-(\frac{k_{2}+k_{3}}{V_{i}})\cdot C_{i}+\frac{k_{4}}{V_{i}}\cdot C_{c}; \end{array}$$ (4)

$$\begin{array}{cc} \frac{dC_{c}}{dt} & =\frac{k_{3}}{V_{c}}\cdot C_{i}-(\frac{k_{4}+{\text{CL}}_{c}}{V_{c}})\cdot C_{c}. \end{array}$$ (5)

The parameters here are defined as follows,

$$\begin{array}{cc} k_{1}= & {\text{F}}_{\text{unbound}}\cdot {\text{P}}_{\text{endothelial}}\cdot {\text{SA}}_{\text{med}\_\text{int}\_\text{liver}},\\ k_{2}= & \frac{k_{1}}{{\text{K}}_{\text{int}\_\text{med}}},\\ k_{3}= & {\text{K}}_{\text{water}\_\text{int}}\cdot {\text{PA}}_{\text{int}\_\text{cell}}\;\text{and}\\ k_{4}= & {\text{K}}_{\text{water}\_\text{cell}}\cdot {\text{PA}}_{\text{cell}\_\text{int}}. \end{array}$$ (6)

where K_int_med_ is the partition coefficient for the transfer of drug between the media and interstitium, P_endothelial_ is the permeability coefficient of the drug between the endothelial layer, SA_med_int_liver_ is the surface area of the interstitium, K_water_cell_ is the partition coefficient for water exchange or movement within the interstitium, K_water_cell_ is the partition coefficient for water exchange or movement within the intracellular compartment, PA_cell_int_ is the product of the surface area and permeability coefficient of the cellular membrane in the intracellular compartment, PA_int_cell_ is the product of the surface area and permeability coefficient of the cellular membrane in the interstitium compartment and F_unbound_ is the fraction unbound (media, reference value). All the relevant variables and parameters with descriptions are given in Table 2.

**Table 2:** Names and description of all relevant variables and parameters in the three-compartment digital twin.

Parameter	Description	Unit	Status
K_int_med_	Partition coefficient for the transfer of drug between the medium and interstitium	dimensionless	calculated
K_water_int_	Partition coefficient for water exchange or movement within the interstitium	dimensionless	calculated
K_water_cell_	partition coefficient for water exchange or movement within the intracellular compartment	dimensionless	calculated
P_endothelial_	The rate of passive diffusion across the endothelial layer, which is the product of surface area and the permeability coefficient of the drug	cm/min	estimated
PA_int_cell_	Permeability coefficient of the cellular membrane in the interstitium compartment	mL/min	calculated
PA_cell_int_	Permeability coefficient of the cellular membrane in the intracellular compartment	mL/min	calculated
F_unbound_	Fraction unbound (medium, reference value)	dimensionless	calculated
SA_med_int_liver_	Surface area of the interstitium	cm^2^	calculated
CL_c_	Intrinsic on-chip clearance	mL/min	estimated
Variables			
*C*_*m*_	Concentration of the drug in the media compartment	*μ*mol/mL	simulated
*C*_*i*_	Concentration of the drug in the interstitium compartment	*μ*mol/mL	simulated
*C*_*c*_	Concentration of the drug in the intracellular compartment	*μ*mol/mL	simulated

We can write this system of linear ODEs in matrix form,

$$\begin{array}{cc} C^{\prime }= & \;A\cdot C,\;\text{where}\\ A= & [\begin{array}{cccc} -\frac{k_{1}}{V_{m}} & \frac{k_{2}}{V_{m}} & 0 & \\ \frac{k_{1}}{V_{i}} & -(\frac{k_{2}+k_{3}}{V_{i}}) & \frac{k_{4}}{V_{i}} & \\ 0 & \frac{k_{3}}{V_{c}} & -(\frac{k_{4}+{\text{CL}}_{c}}{V_{i}}) \end{array}]. \end{array}$$ (7)

The general solution for the system of ODEs at time *t* is:

$$\begin{array}{c} C(t)=e^{A\cdot t}=e^{X\cdot \Lambda t\cdot X^{-1}}\cdot C_{0}=X\cdot e^{\Lambda t}\cdot X^{-1}\cdot C_{0}, \end{array}$$ (8)

where *X* is the matrix of eigenvectors of *A*, Λ is the diagonal matrix with λ_1_, λ_2_, λ_3_ (eigenvalues of *A*) as diagonal entries, and *C*_0_ is the initial value of variables at time 0.

The objective function for optimisation is as follows:

$$\begin{array}{c} \underset{{\text{CL}}_{\text{c}}}{\text{minimise}}\sum_{i=1}^{n}{\left(y_{i_{\text{obs}}}-y_{i_{\text{a}}}\right)}^{2}\{\begin{array}{cc} & y_{i_{\text{a}}}=e^{A\cdot t_{i}}\cdot C_{0}\\ & \text{computed}\;\text{using}\;\text{eigenvalues}\\ & \text{and}\;\text{eigenvectors,} \end{array} \end{array}$$

where $y_{i_{\text{obs}}}$ is the observed data point and $y_{i_{\text{a}}}$ is the computed value at time *i* respectively and *n* is the number of observed data points.

### 2.3 DigiLoCs: Digital twins for cell-based liver assays

**Fig 1:**
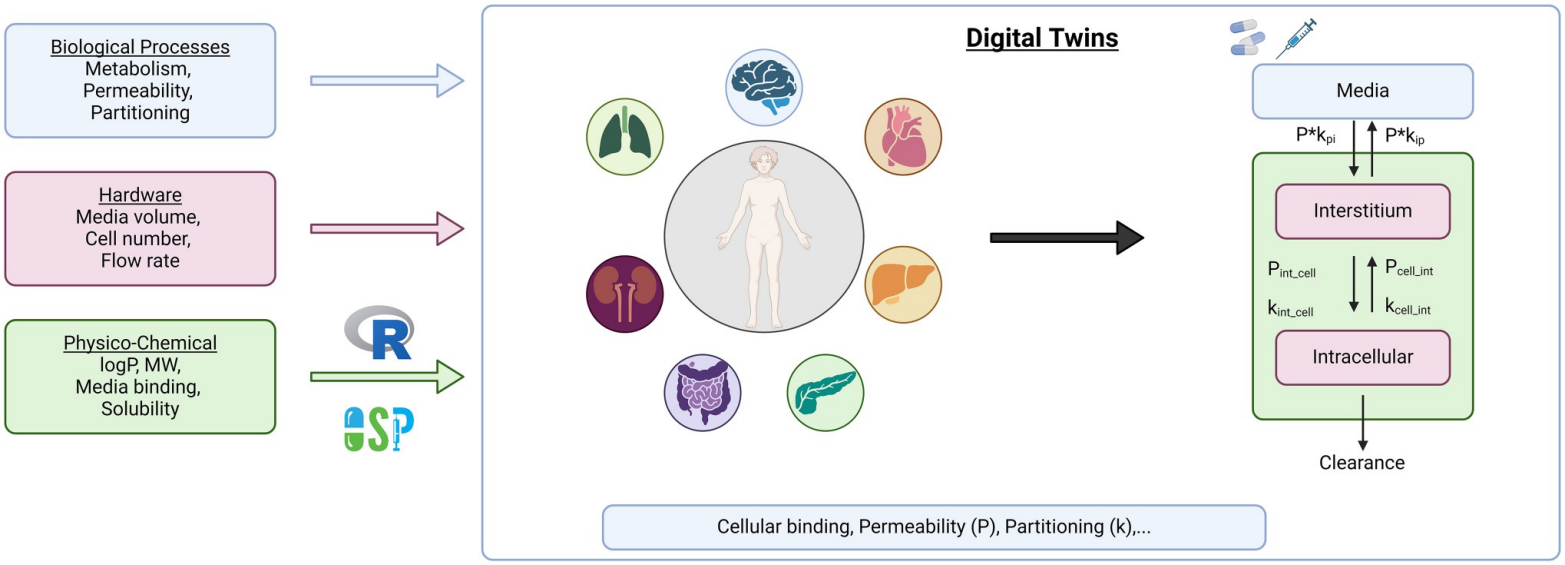
Digital Twin (DT) approach. Contrasting conventional approach, the DT approach uses biological, hardware, and physicochemical information to map the biological processes on liver-chip more accurately to *in silico*, thereby maximising the information leveraged. This results in the disentanglement of active (metabolism) and passive (permeability, partitioning) processes. Created with biorender.com.

DigiLoCs is a software tool, developed within this work that describes the on-chip complex biology more accurately in the context of use to predict clinical clearance values. The software comprises (Fig 1):

- modelling of complex biological processes (clearance, permeability, partitioning),
- hardware-specific information from the studied *in vitro* system and
- compound-specific information.

The tool differentiates between active biological processes, such as metabolism, and passive ones, like permeability and partitioning of a compound from cell culture media into the cellular milieu. This contrasts with conventional approaches, where passive biological processes are not considered especially and lumped together into a single process, i.e., clearance.

Liver-on-chip technology provides a more physiologically relevant environment compared to traditional cell cultures or animal models, enhancing the simulation of drug responses using mathematical models. Hence, a more accurate mathematical description is needed. The three primary compartments of the liver chip considered in the model are media, interstitium, and intracellular space, which serve as dynamic environments where drugs are distributed, metabolised, and interact with hepatic cells. This compartmentalisation is based on concepts applied in human whole-body PBPK modelling.

The software tool is developed in the open-source programming language R [21] and seamlessly communicates with PK-Sim (https://www.open-systems-pharmacology.org/) via in-house developed functions. For more information, see esqlabsR package (https://github.com/esqLABS/esqlabsR). All analysis and plotting were also done in R. The proposed workflow does not interfere with existing wet lab Standard Operating Procedures (SOPs) for performing biological experiments and does not add an extra considerable burden to the user. DigiLoCs uses existing biological data, and its performance may be improved by measuring cell-associated compound concentrations in addition to the compound media depletion time course, which would add a minor extra step in the lab SOP. This, however, is negligible given the improvement in performance power and the confidence in the prediction.

#### 2.3.1 Implementation of hardware specifications

DigiLoCs map the chip architecture to a compartmental model to describe the time-dependent distribution of a compound on-chip. The compartment models use time-dependent ordinary differential equations (ODEs) and assume well-mixing within compartments. These are generally accepted to describe the distribution of exogenous and endogenous compounds and molecules.

A physical chamber separated by a membrane or connected by flow to another chamber is represented by a compartment in the software. Serial compartments are connected via media flow rates (typically in *ml*/*min*) between the compartments describing mass transport via gradients and diffusion and normalised by the volume of the originating compartment.

#### 2.3.2 Implementation of biological specifications

The biology (more precisely, the cell type exerting the biological function under investigation; here: metabolism) is mapped by two additional compartments representing the interstitial and intracellular space of the investigated biology (Fig 1). Transport rates from the cell culture media into the interstitial and intracellular milieu are described by two core processes:

- passive diffusion (driven by concentration gradients between compartments)
- thermodynamic equilibrium (distribution of compound between two phases)

These processes are then described by two main parameters in the computational model: permeability (how fast is a compound taken up?) and partitioning (how much of the compound is taken up by cells?). Lastly, the metabolism rate is reallocated to the intracellular compartment and corrected by the unbound fraction of the compound in the intracellular compartment. Similarly, in pharmacokinetics, the distribution of compounds is often described using a compartmental framework, which involves dividing a system into distinct compartments and modelling the processes that govern the movement of compounds between them.

#### 2.3.3 Implementation of compound-specific information

The following physicochemical properties of the investigated compounds are used in the software: *i*) lipophilicity (logP), *ii*) molecular weight (MW) and *iii*)fraction unbound (fu); to calculate up to six dependent downstream parameters (listed below). These parameters describe the partitioning from the main media compartment into the interstitial space and between the water fraction and both interstitial and intracellular space. Additionally, permeability across the endothelial barrier and between interstitial and intracellular spaces are calculated.

Partitioning: *i*) K_int_med_ *ii*) K_water_cell_ *iii*) K_water_cell_
Permeability: *i*) P_endothelial_ *ii*) PA_cell_int_ *iii*) PA_int_cell_

Here *int* refers to interstitial space, *med* refers to media, *water* refers to water exchange fraction, and *cell* refers to intracellular space. These parameters are calculated based on well-established and documented equations implemented in PK-Sim software [3], which is a comprehensive software tool for whole-body PBPK modelling. It enables rapid access to all relevant anatomical and physiological parameters for humans and common laboratory animals contained in the integrated database for model building and parameterisation. The same partition coefficient calculation methods as implemented in PK-Sim are also readily available and can be investigated:

- PK-Sim standard
- Poulin and Theil
- Rodgers and Rowland
- Schmitt
- Berezhkovskiy

Further, only the unbound fraction of a compound can be taken up by cells and be metabolised by cells. The unbound fraction in the cell culture media is typically informed by biological experiments. However, the intracellular unbound fraction is not often available or measured. Thus, two established QSAR models (quantitative structure-activity relationship) are implemented in the software to predict the unbound intracellular fraction of the investigated compound as a function of its physicochemical properties [22, 23].

### 2.4 Parameter estimation

Parameter estimation aims to find unknown parameters in a computational model and is estimated using experimental data collected from well-defined and standard conditions. By minimising the distance of theoretical function values and experimentally known data, the set of parameters in the model can be estimated. The parameters which are not directly measurable can be estimated using least squares or any other fitting methods to analyse the model quantitatively. Nominal parameter values are obtained from PK-Sim software.

Parameter estimation in DigiLoCs is a two-step process. Firstly, a customised cost function is implemented. This cost function calculates the weighted difference (ssq) between the model simulation (pred) from a specific compartment and the corresponding observed data (obs) for each time point according to the equation

$$\begin{array}{c} \text{ssq}=\frac{\text{obs}-\text{pred}}{\text{pred}}. \end{array}$$ (9)

Common parameter estimation methods include maximum likelihood estimation and Nelder Mead optimisation. Nelder Mead, a non-linear optimisation method, is used to find the minima of the objective function in this work. Additionally, the partition coefficient between the intracellular (IC) and the main media compartment is estimated using the area under the simulated time-concentration profile of the IC and interstitial (IST) compartment and corrected for by the QSAR-predicted cellular unbound fraction (fu_cell_) and the unbound fraction in the media (measured, fu_media_):

$$\begin{array}{c} {\text{Kp}}_{\text{uu},\text{pred}}=\frac{\text{AUC}(\text{IST}+\text{IC})}{\text{AUC}(\text{media})}\cdot \frac{{\text{fu}}_{\text{cell}}}{{\text{fu}}_{\text{media}}}. \end{array}$$ (10)

Kp_uu,obs_ is calculated from literature [24], where media and intracellular concentrations in hepatocytes were determined. Initially investigated for suspension hepatocytes in a 2D setting, the authors provide a scaling factor (∼4.9) to apply to human hepatocytes. Further, the ionisation state of the investigated compound (-1, 0, 1) results in a different partitioning. Otherwise, a range of possible partition coefficients are investigated. This is an additional anchor point for estimating the cost function value and links the simulated intracellular and main media compartment concentrations. Eventually, both differences are squared and summed up, resulting in the final sum of residuals. Based on this, a compound-specific scaling factor (SF) is calculated and used to scale the predicted human clearance:

$$\begin{array}{c} \text{SF}\left(\text{drug}\right)=\frac{{\text{Kp}}_{\text{uu},\text{obs}}}{{\text{Kp}}_{\text{uu},\text{pred}}}. \end{array}$$ (11)

Specifically for the liver use cases, on-chip liver clearance and surface area between the main media compartment and the cell layer are estimated. It is possible to estimate other parameters, such as pre-calculated permeability or partition coefficient values.

#### 2.4.1 Implementation of software

Methodologically, DigiLoCs is implemented in the open-source programming environment R with its own package. A library of two common chip architectures and two cell types with six different chip-specific settings have already been implemented.

1. One chamber, no media flow
2. Two chamber, recirculating flow
3. Organ-on-chip (hepatocytes)
  a. CnBio
  b. Hurel 1
  c. Hurel 2
  d. Dynamic42
  e. Javelin

**Fig 2:**
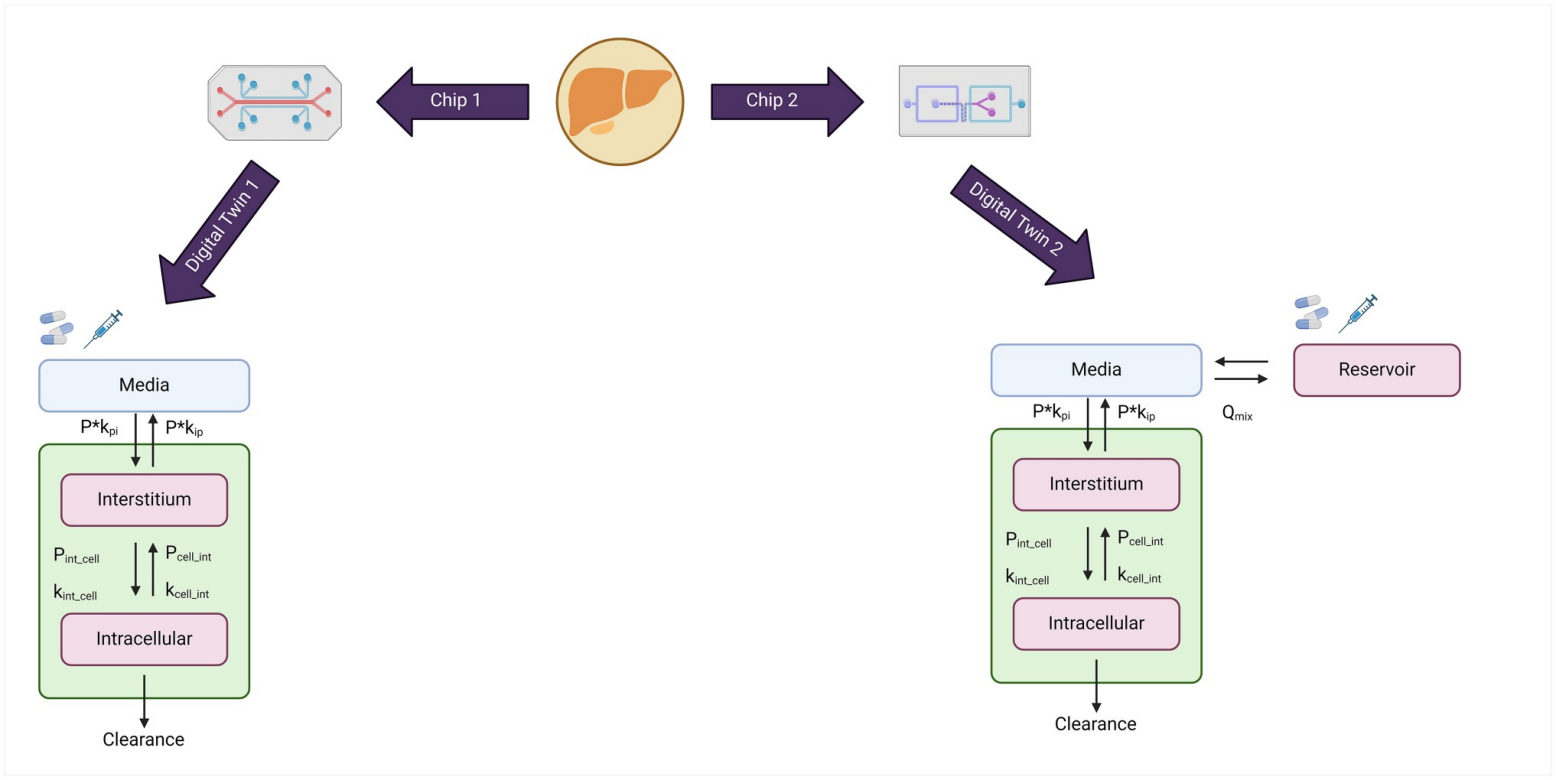
Chip 1 is for CnBio [13] and 3D spheroids [28] and chip 2 is for Javelin [20] architectures. Q_mix_ is the mixing flow rate in mL/min. Created with biorender.com.

The most straightforward system is a single, perfused microfluidic chamber containing one kind of cultured cell (e.g., hepatocytes) that exhibits functions of one tissue type linked to channels for fluid transport. In more complex designs, two or more microchannels are connected by porous membranes, lined on opposite sides by different cell types, to recreate interfaces between different tissues. The CnBio and Javelin chip settings are shown in Fig 2. For detailed information on the processes and modelling, readers are directed to Bhatia et al. [25]. These building blocks can be interchangeably used and connected, similar to the building blocks in PK-Sim. While the package provides a step-by-step guide to generate and run a simulation, the code communicates seamlessly with a generic PK-Sim model to determine partitioning and permeability values as described above, which are used in the simulation.

### 2.5 Sensitivity analysis

Both local and global sensitivity analyses are used to quantify the impact of input parameters on the output variables. This involves varying certain input parameters and observing changes in the output variable intracellular concentration. The local sensitivity is estimated by changing one input parameter at a time while other parameters are held constant. The study provides insights into the sensitivity of various parameters and how they affect the output of the model.

The input parameters K_int_med_, P_endothelial_, SA_med_int_liver_, K_water_int_, K_water_cell_, PA_cell_int_, PA_int_cell_, fu and CL_*c*_ are varied to evaluate the sensitivity of the output variable intracellular concentration (*C*_*c*_). First, the local sensitivity is estimated by changing one input parameter by 10% at a time while the other parameters are held constant, and the changes in output variable *C*_*c*_ are compared. Eq 12 shows the local sensitivity index for *C*_*c*_ with respect to the varying model parameter (*P*_*i*_), which is approximated by a small perturbation Δ*P*_*i*_,

$$\begin{array}{c} \frac{\delta C_{c}}{\delta P_{i}}=\underset{\Delta P_{i}\to +0}{\text{lim}}\frac{C_{c}\left(P_{i}+\Delta {\text{P}}_{i},P_{n\neq i}\right)-C_{c}\left(P_{i}\right)}{\Delta P_{i}}, \end{array}$$ (12)

where *C*_*c*_(*P*) is the model prediction of the intracellular concentration for parameter set *P*. The local sensitivity index is normalised to eliminate the effect of units,

$$\begin{array}{c} S_{i}=\frac{\delta C_{c}}{\delta {\text{P}}_{i}}\frac{{\text{P}}_{i}}{C_{c}\left(\text{P}\right)}. \end{array}$$ (13)

Global sensitivity analysis evaluates the effect of potential interactions of the input parameters in an output variable. The Sobol sensitivity analysis of the SALib package in Python is used to perform the global sensitivity analysis. Input parameters are sampled using the Saltelli sampler. The lower and upper bound of the parameters are set as 0.1-fold and 10-fold of the baseline parameter values, respectively. The first-order and total-order indices are estimated using the Sobol sensitivity analysis.

### 2.6 Translation to humans

Drug-related parameters extracted from OoC or any other *in vitro* studies can be scaled to predict clinical parameters using *in vitro*-*in vivo* translation (IVIVT) [13, 17]. The typical value of unbound intrinsic clearance CL_int(u)_ determined for each drug from the pharmacokinetic analysis of the *in vitro* depletion data is scaled up to a human liver equivalent unbound intrinsic clearance CL_int(u),(H)_ using

$$\begin{array}{c} {\text{CL}}_{\text{int}(\text{u}),\text{H}}=\frac{{\text{CL}}_{\text{int}(\text{u}),\text{H}}\cdot \text{HC}\cdot \text{LW}}{{\text{fu}}_{\text{inc}}}, \end{array}$$ (14)

where HC is the human hepatocellularity of 120 million cells / g of liver, LW is the average human liver weight of 25.7g / kg of body weight [17] and fu_inc_ is the unbound fraction of drug in the incubation medium. The hepatic clearance (referring to whole blood concentrations) is then predicted (CL_H,pred_) using the Well-Stirred (WS) model:

$$\begin{array}{c} {\text{CL}}_{\text{H}}\left(\text{pred},\text{WS}\right)=\frac{{\text{Q}}_{\text{H}}\cdot {\text{fu}}_{\text{b}}\cdot {\text{CL}}_{\text{int}(\text{u}),\text{H}}}{{\text{Q}}_{\text{H}}+{\text{fu}}_{\text{b}}\cdot {\text{CL}}_{\text{int}(\text{u}),\text{H}}}, \end{array}$$ (15)

where Q_H_ is the average hepatic blood flow of 20.7 mL/min/kg of body weight and fu_b_ is the fraction of the drug unbound in blood. The fraction unbound in the blood was calculated for each compound from the known fraction unbound in the media (fu_p_) and blood-to-plasma ratio (Rbp) according to the equation fu_p_ = fu_p_/Rbp or directly used, if available from the literature. The predicted hepatic clearance (CL_H,pred_) values were then compared to observed hepatic clearance (CL_H,obs_) values (referring to whole blood concentrations). Following, the ratio of predicted (either via conventional or digital twin approach) and observed clinical clearance values for all investigated compounds was calculated. Using these ratios, a density distribution function was computed (function geom_density from ggplot [26]) for visual purposes only.

### 2.7 Prediction of human pharmacokinetics

Initially, a PBPK model is developed using qualified installations of the software PK-Sim that ensures the software has been installed correctly and thoroughly validated according to established procedures. A whole-body PBPK model includes an explicit representation of the organs most relevant to the uptake, distribution, excretion, and metabolism of the drug. These typically include the heart, lungs, brain, stomach, spleen, pancreas, intestine, liver, kidney, gonads, thymus, adipose tissue, muscles, bones, and skin. More information can be found in S1 File.

**Fig 3:**
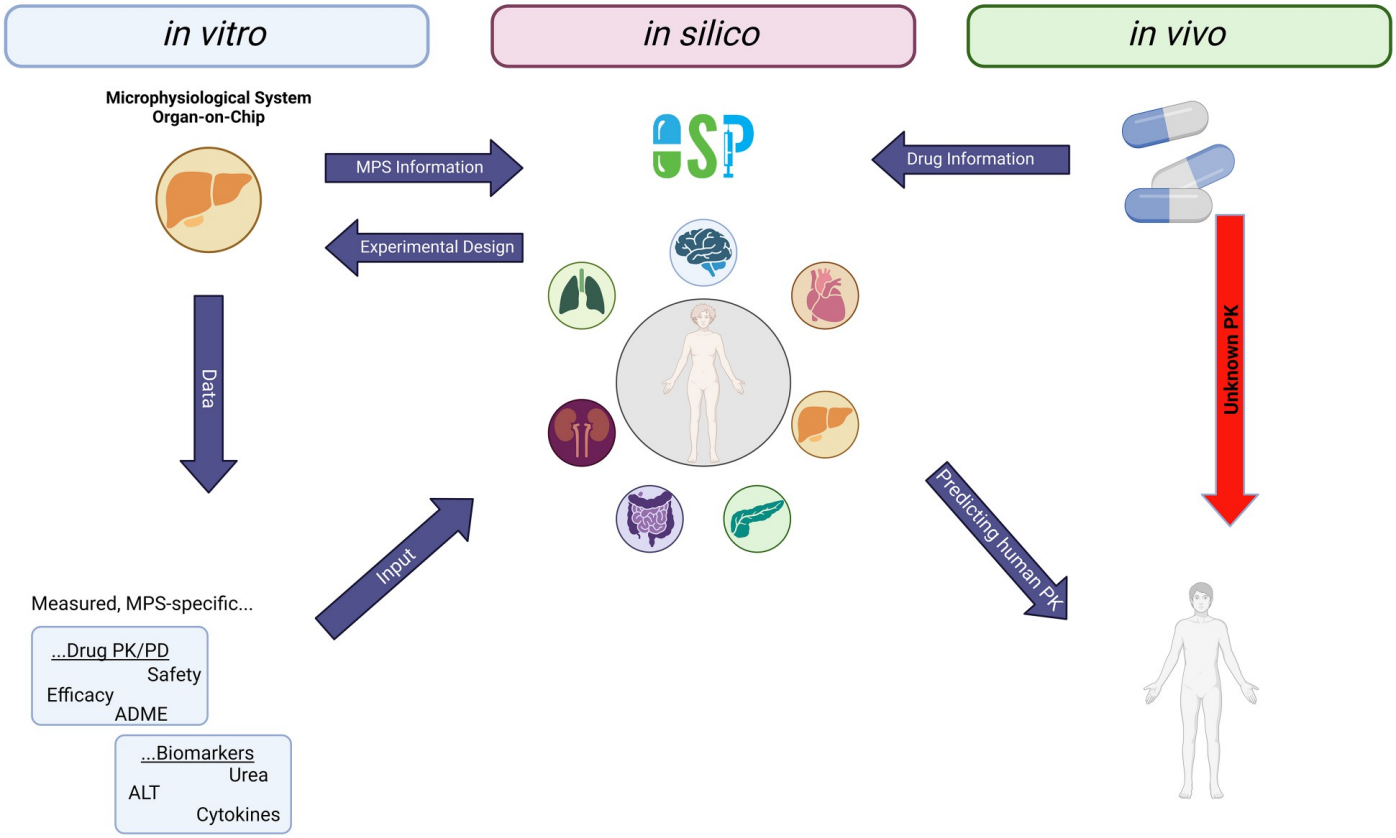
Translational workflow plan that integrates results from organ-on-chip with computer modelling to predict the kinetics of drugs in humans. The digital twins of the humanised organ-on-chip systems, together with chip-specific information and physicochemical information, are developed in R. Created with biorender.com.

The tissues are interconnected by arterial and venous blood compartments, and each is characterised by an associated blood flow rate, volume, tissue partition coefficient, and permeability. If applicable, R (Distribution 4.0) and RStudio (Version 1.2.5) are used in the analysis for preprocessing and post-processing of data and model outputs [27]. The analytical approach is based on the principles set out in the EMA, FDA, and/or OECD guidelines for reporting on PBK M&S [11]. The developed PBPK model is used to describe the human kinetics of propranolol. Key kinetic parameters are informed by either clinical data, literature values or on-chip predictions. The translational workflow that integrates organ-on-chip results to predict human pharmacokinetics is shown in Fig 3.

## 3 Results

We report the following results in this section. First, results from a simulation model based on a digital twin for selected compounds; second, sensitivity analyses; third, prediction of human clearance values; and finally, translation to human PK using propranolol as a proof-of-concept study. The liver clearance and surface area of the chip are estimated after fitting the drug kinetic data. The Poulin and Theil method of partition coefficient calculation was used due to its superior fit to observed drug kinetics, which outperformed alternative methods.

### 3.1 Simulating on-chip compound depletion

**Fig 4:**
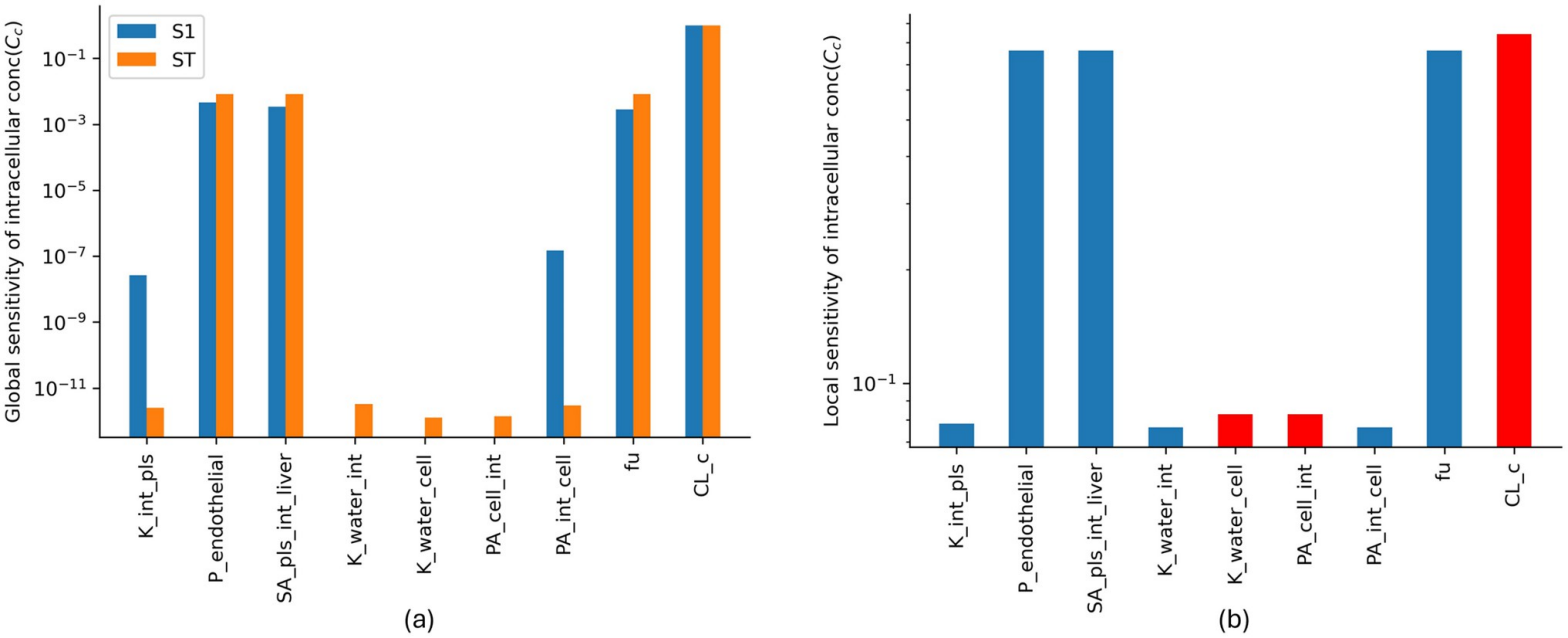
Digital twin-based model simulation of on-chip kinetics after fitting parameters for selected compounds. Observed data are shown in blue dots, where the data for diclofenac, midazolam, and oxazepam are from Docci et al. [13], while propranolol is from Tsamandouras et al. [17]. The red, violet and green curve plots the drug concentration in the intracellular, medium and interstitium compartments, respectively: IC = intracellular, Ist = interstitium.

The digital twins for the investigated *in vitro* liver systems were successfully implemented in R and used to simulate the on-chip kinetics. After parameter estimation, the resulting model simulations describing the observed compound depletion data were visually inspected. The final parameter values can be found in Tables A and B in S1 File. Additionally, the squared sum of residuals was evaluated and deemed acceptable as it was less than < 0.01, which was the case for all simulations (data not shown). An example of on-chip kinetics is presented in Fig 4.

As can be seen, the digital twin approach (violet line) captures the on-chip kinetics (blue dots) very well. Simultaneously, the intracellular (IC) kinetics are plotted (red lines), clearly highlighting the difference in compound uptake and, thus, clearance rates. The remaining figures are presented in S1 File (See Figs A-D).

### 3.2 Sensitivity analysis

The sensitivity analysis, both local and global, was conducted to quantify the sensitivity of model output intracellular concentration with input parameters. The analyses were performed for various parameters, and the results indicated that the output is more sensitive to parameters such as the permeability coefficient of the endothelial layer, surface area of the liver sinusoids, and clearance. These parameters were estimated or calculated from clinical data/ experimental results. Clearance (CL_*c*_) is identified as the most sensitive parameter with respect to intracellular concentration. The results imply that accurate values of these sensitive parameters are crucial for the model’s accuracy.

**Fig 5:**
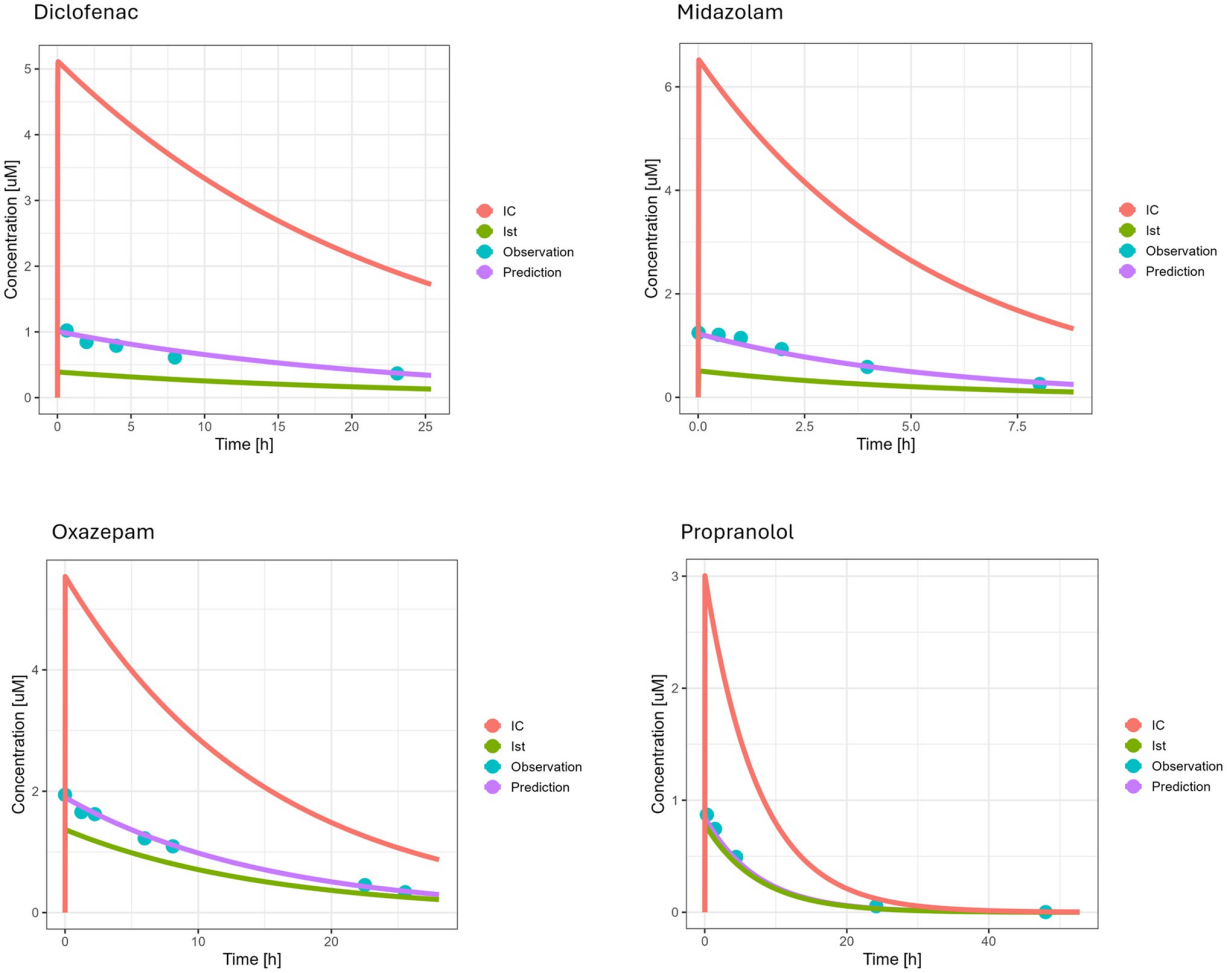
Local and global sensitivity of the parameters with respect to output intracellular concentration. (a) Blue bars indicate that the output and the input changes in the same direction, and the red bar indicates that the output decreases when the input increases. (b) The blue and orange bars represent first-order and total-order indices, respectively.

The normalized local sensitivity indices (Fig 5a) and the first-order and total-order global sensitivity indices (Fig 5b) for intracellular concentration across the input parameter set are shown. The results from both local and global sensitivity analyses show that the output is more sensitive to parameters P_endothelial_, SA_pls_int_liver_, fu and CL_*c*_. SA_pls_int_liver_ and CL_*c*_ were estimated and nominal values were used for all other parameters. SA results imply that we need correct values of the constants P_endothelial_, fu as they are more sensitive.

### 3.3 Predicting human clearance

The on-chip estimated clearance values were translated to total human clearance according to Eq 15. More detailed information is available in S1 File (see Tables A and B). Likewise, from the investigated studies (Table 1), *in vitro* unbound clearance values were available and scaled to human equivalents.

**Fig 6:**
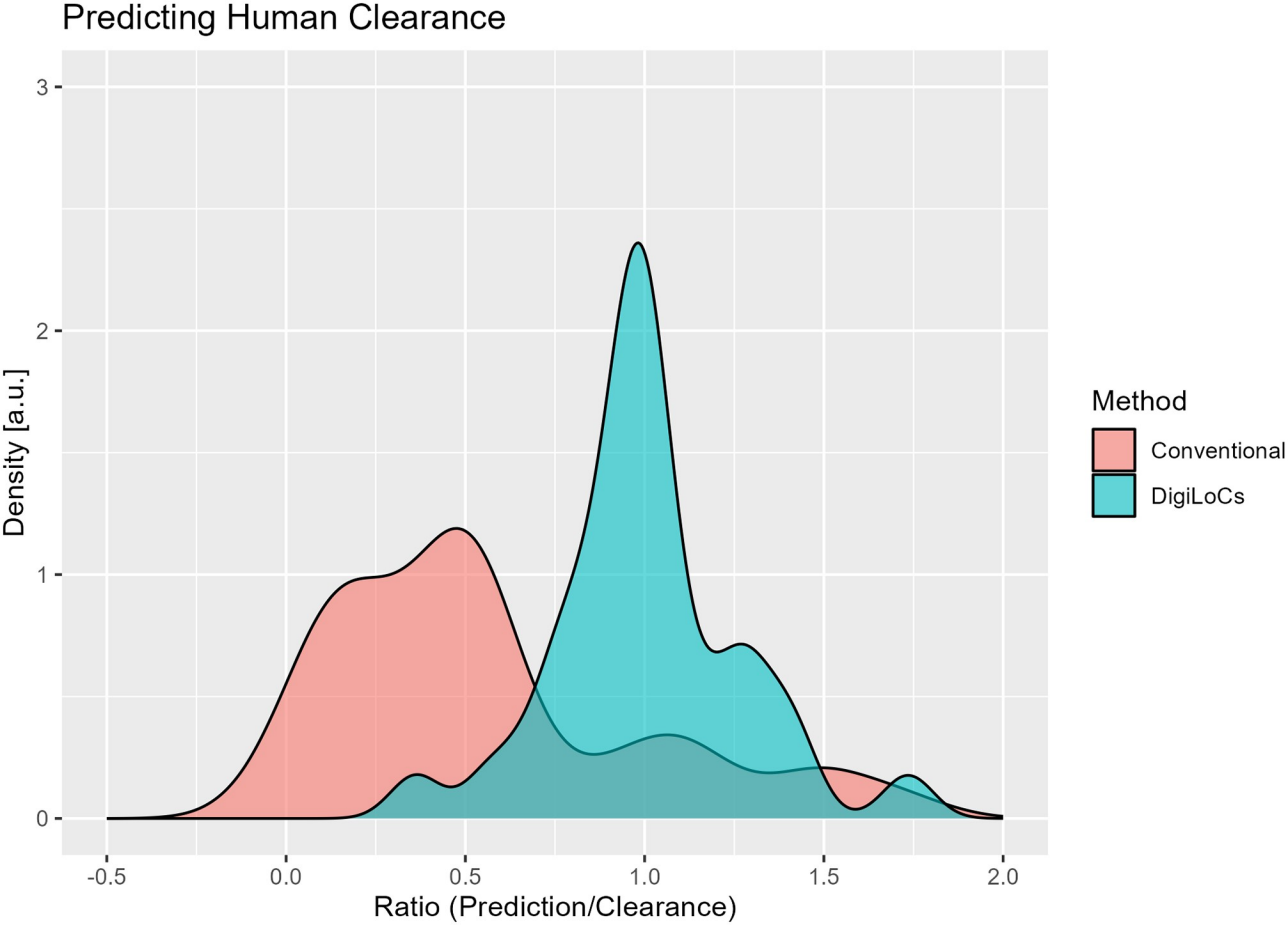
Impact of DigiLoCs on predicting clinical clearance values compared to the conventional approach. In total, a set of 32 compounds across three different *in vitro* liver systems have been investigated. The x-axis presents the ratio of predicted/observed clinical clearance values using either the DigiLoCs or the conventional approach, and the y-axis shows the frequency of the ratio observed.

**Fig 7:**
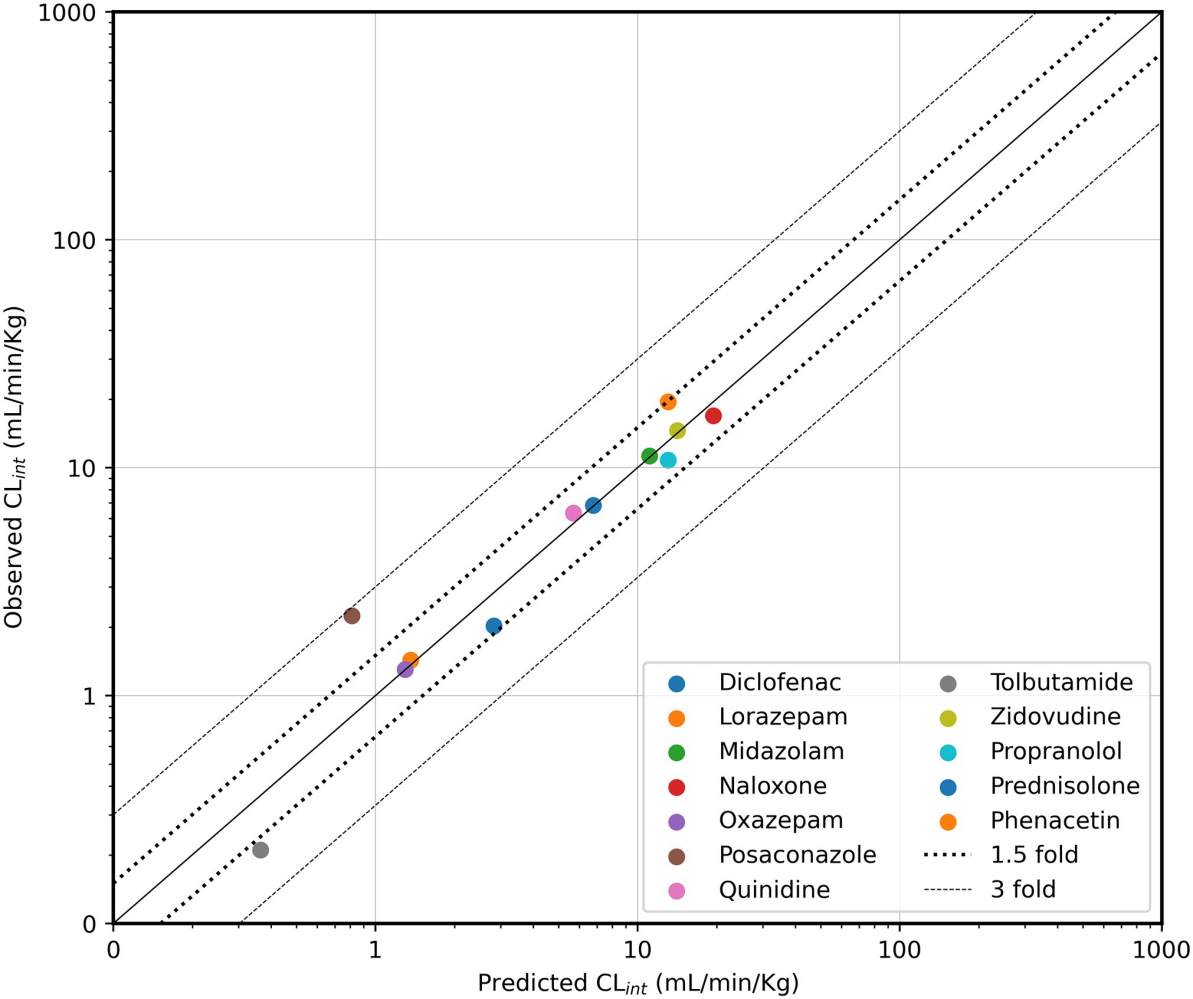
Correlation between observed and predicted *in vivo* intrinsic clearance (CL_int_) using the three-compartment liver chip for 12 drugs (Docci et al. 2022; Tsamandouras et al. 2017). The solid line shows the line of unity, while the dotted line is 1.5-fold, and the dashed line has a 3-fold deviation.

The conventional approach is a single-compartment model explained in Section 2.2 using a single ODE. The ratio of clinical observed human clearance values and either predicted human clearances using conventional mathematical modelling or the digital twin approach were estimated and converted into a density function for easier graphical visualisation as described in Section 2.6. As can be seen in Fig 6, the digital twin approach (DigiLoCs) outperforms the conventional approach considerably. The centre of the distribution is around 1, indicating a non-biased prediction of clinical clearance values, while the width of the distribution is very small. Quantitatively, the ratio for the digital twin approach overall compounds is 1.04 ± 0.31, with a coefficient of variation of 30%. In contrast, the conventional approach (red curve) majorly under-predicts the clearance values while maintaining a broad distribution and thereby adding to uncertainty in the prediction (0.56 ± 0.44, CV = 79.3%). The correlation plot between the observed and herein predicted clinical clearance values highlights the improved prediction performance of the DigiLoCs approach on an individual drug level. As can be seen in Fig 7 (example graph for CnBio Liver-on-Chip data), most of the compounds fall within the 1.5-fold line (Average fold error, AFE = 0.965). Similar correlation plots are presented in the S1 File (See Figs E-G) for the other *in vitro* systems.

### 3.4 Translation to human PK

The impact of accurately predicting human clearance values based on *in vitro* cell-based assays on predicting human PK was assessed using propranolol as a proof-of-concept case study. First, a human PBPK model describing the human kinetics of propranolol was implemented in PK-Sim and qualified with clinical observations. Next, the predicted human clearance value using either the conventional modelling approach or based on the same on-chip kinetic data was implemented in the human PBPK model simulating the kinetics after a single oral dose. Further, a population of *n* = 1000 patients was simulated to account for inter-patient variability. As shown in Fig 5, the implemented human PBPK model describes observed clinical data well (using clinical clearance values). When substituting only the clearance value with the conventional or the digital twin-based values, the impact on simulating human PK becomes apparent, while the conventional approach would overpredict (i.e., the on-chip clearance is underpredicted) the human PK (3-fold C_max_, up to 6-fold overprediction of AUC). Moreover, this approach would actually simulate non-negligible concentrations of propranolol left over after 24 h. For repeated daily dosing, this would result in accumulation of propranolol in this hypothetical setting, which would have immediate implications for potential toxicity or efficacy considerations. On the other hand, the digital-twin based approach still slightly overpredicts the AUC and C_max_, however only by 1.5-fold and captures the terminal phase correctly.

## 4 Discussion

The aim of this work is to improve the current prediction of human clearance values and to present a framework for translating *in vitro* findings to relevant clinical situations. The presented integrated translational approach combined quantitative OoC and cell-based assay compound depletion kinetics with an OoC-digital twin to simulate drug kinetics in humans.

Initial investigations revealed the potential to describe clinical clearance values more appropriately than is currently possible with the conventional approach. This simpler approach lumps biological processes together into a single process—clearance—and uses only minimal information available, e.g., only the cell number and media volume. While biological systems have evolved rapidly in the last decade, especially in the field of organ-on-chip and microphysiological systems, the applied mathematical models to analyse the quantitative complex biological data have been the same for decades (the early concept of clearance was introduced by Möllers in 1928, while Well-stirred model was introduced in 1971).

In contrast, the digital twin approach for the organ-on-chip and 3D spheroids comprises three building blocks described here: biological, hardware, and physicochemical information. The distinction between active and passive processes is achieved by an explicit description of uptake, distribution, and metabolism involved in the biological processes. Further, the digital twin links the architecture of the hardware chip with an advanced mapping of the underlying biology (intracellular compartment). The on-chip kinetics for 32 compounds (six compounds were removed from the initial set due to missing information) was well described, highlighting the drug-specific effects on cellular uptake and hence metabolism. Note that this analysis used the same biological information as used in the conventional approach and that no additional biological experiments were needed or performed to improve the outcome of the digital twin approach.

The predictive power of organ-on-chip and 3D spheroids over conventional approaches was revealed when the depletion data was analysed with the digital twins (Fig 6). Not only was the systematic underprediction issue resolved, but the uncertainty in prediction was also reduced by a factor of 3 (comparing CVs).

**Fig 8:**
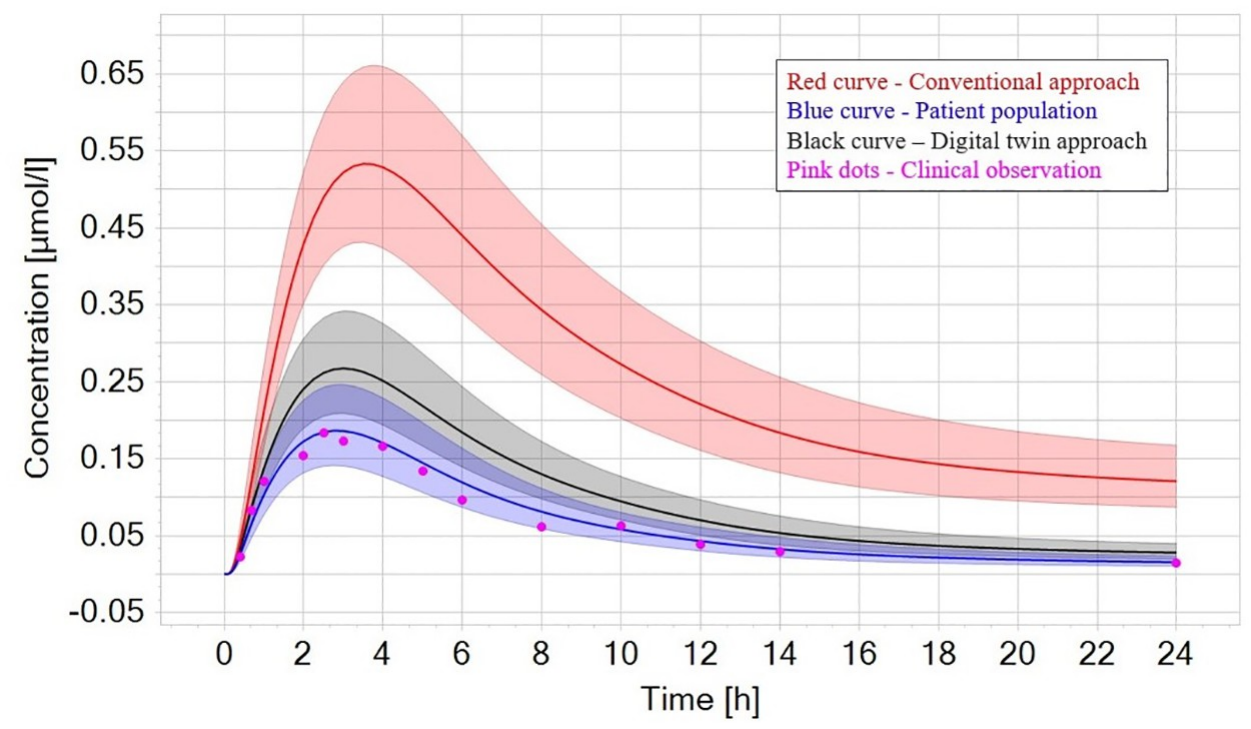
Simulated kinetics of propranolol after a single oral dose (80 mg). Pink dots are clinical observations (digitised from Borgström et al. [29]), while the blue solid line represents the mean of the patient population using the clinical observed clearance value. When using the clearance value (red line) based on conventional approaches, the area under the curve is 6-fold overpredicted. In contrast, using the digital twin-based clearance, the AUC is only 1.5-fold overpredicted, also simulating the right kinetics at 24 h (black curve). Shaded areas represent ± 1 SD.

Lastly, we aimed to demonstrate the clinical impact of this approach by translating the results to humans using propranolol as a proof-of-concept example. Here, the head-to-head comparison clearly demonstrated the superior power of both quantitative biological data from OoCs and digital twins over conventional approaches in predicting human PK more appropriately (Fig 8). Although only one compound was used to demonstrate clinical impact, the workflow and process are easily applicable to other compounds. To the best of our knowledge, this study is the biggest comprehensive report to systematically assess the predictive power of organ-on-chip in the context of use of liver clearance.

The mathematical algorithm to determine liver clearance depends on the time-concentration profiles and, if available, on intracellular or cell-associated compound concentrations. The algorithm minimises a cost function by identifying a clearance value such that model prediction and data observation match. The cost function takes both the PK profile from cell culture media and the cell-associated levels into account, which is not the case for the conventional approach. Further, binding of the compound to plastic/hardware of the chips, to proteins contained in the cell culture media, or any other intracellular lipids can be accounted for to accurately determine liver clearance. DigiLocs, further, also does not depend on a scaling factor, which overcomes the systematic underprediction of conventional approaches (5–10 fold on average across multiple studies).

So far, limited information is available from the literature or in-house measurements on the observed partitioning of compounds into the intracellular or cell-associated milieu of hepatocytes. If that data becomes available, it may be incorporated compared to the adjustments made in the software to match the clinical clearance values. If the predicted and observed Kp_uu_ values match, the digital twin approach truly improves the prediction. If there is a discrepancy between these values, the fitting process can be re-run including the observed Kp_uu_ value. This would inform the maximum capacity of the system to metabolise the compound. If this final rate is still lower than the observed clinical clearance value, two options are possible to understand and improve the prediction:

1. Calculate a correction factor, which is compound-specific and chip-specific and not generic like in the conventional approaches.
2. Investigate other model-specific parameters to optimise, e.g., permeability or partitioning.

Although initially developed for hepatic clearance, the mathematical model can be employed for toxicity or efficacy-related questions depending on the context of use. In such a setting, time-concentration profiles will be simulated and linked to other measured biomarkers (e.g., ATP (adenosine triphosphate), TEER (barrier integrity)) to determine IC50 or EC50 values, and parameters to assess toxicity and efficacy, respectively. Likewise, the same integration of complex biological processes, hardware-, and drug-specific information can be used to model other cell and chip types, e.g., a blood-brain-barrier-chip, which is used to determine the permeability of compounds across the barrier.

Eventually, we envision DigiLoCs to support the pharmaceutical decision-making process by reducing animal testing and ultimately streamlining the drug development process.

## 5 Conclusion

The development of digital twins for organ-on-chips, reported here, incorporating systems of differential equations-based models and leveraging published data, holds great potential to enhance our understanding of drug behaviour and clinical outcomes. The *in vitro* liver clearance for 32 drugs was predicted using DigiLoCs and a proof-of-concept (translation to human pharmacokinetics) study on propranolol was done. DigiLoCs are envisioned to serve as a decision-support tool for pharmaceutical research, aiding in estimating first-in-human doses, evaluating human pharmacokinetics, and importantly, diminishing reliance on animal experimentation, thereby fostering more efficient, expedited, and sustainable drug development processes. Our approach is generalisable across various physiological contexts and not limited to liver metabolism but may be extended to other organs as well, such as gut metabolism and barrier models such as the brain or placenta.

## Supporting information

S1 File SUPPLEMENTARY INFORMATION FOR DigiLoCS: A leap forward in predictive organ-on-chip simulations. (PDF)
