# Supplementary material for: DigiLoCS: A leap forward in predictive organ-on-chip simulations
Source: PLoS One. 2025 Jan 9;20(1):e0314083. doi: 10.1371/journal.pone.0314083 (PMC11717216; doi:10.1371/journal.pone.0314083)
Supplement: S1 File — (PDF) [file pone.0314083.s001.pdf]

SUPPLEMENTARY INFORMATION FOR
DigiLoCS: A Leap Forward in Predictive Organ-on-Chip Simulations

Manoja Rajalakshmi Aravindakshan[1], Chittaranjan Mandal[1], Alex Pothen[2], Stephan Schaller[3] Christian Maass[3,4*]

1 Department of Computer Science and Engineering, Indian Institute of Technology Kharagpur, West Bengal, India
2 Department of Computer Science, Purdue University, West Lafayette, Indiana, United States
3 ESQlabs Gmbh, Saterland, Germany
4 MPSlabs, ESQlabs Gmbh, Saterland, Germany

*christian.maass@esqlabs.com (CM)

Supporting information Text

The primary objective of this study was to develop an innovative digital twin framework that integrates Microphysiological Systems (MPS) and Organ-on-Chip (OoC) data into advanced computational models of biology to enhance the accuracy of predicting clinical clearances. The resulting tool, known as DigiLoCs, serves as a digital liver-chip simulator capable of effectively capturing the complexities of on-chip biology. It incorporates information on various biological processes such as clearance, permeability, and partitioning, along with hardware-specific details from the studied *in vitro* systems and compound-specific data.

DigiLoCs enables the differentiation between active biological processes like metabolism and passive ones such as permeability and partitioning, which contrasts with existing approaches where passive processes are often lumped together into a single clearance process. The tool's performance was evaluated using drug depletion kinetics data from 32 compounds sourced from literature covering commercially available liver-chips and 3D spheroids. Results indicate that DigiLoCs significantly outperforms current prediction approaches. Furthermore, a proof-of-concept study involving propranolol demonstrated the tool's potential in predicting human pharmacokinetics (PK) more accurately compared to state-of-the-art and literature-based approaches.

Simulation of drug depletion data

The digital twin-based model simulation of on-chip kinetics of all the drugs involved in the study are shown in Figs A-D.

Analysis

The drug-specific information are shown in Table A and the analysis of estimated parameters are shown in Table B. The correlation plots for different *in vitro* systems along with average fold error (AFE) are are shown in Figs E-G.

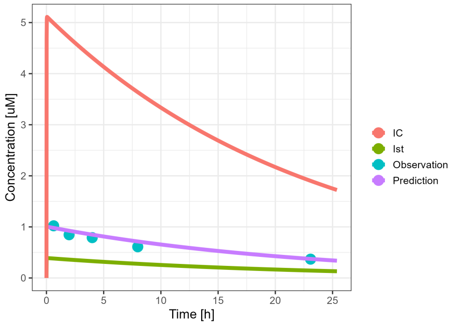

(a) Diclofenac

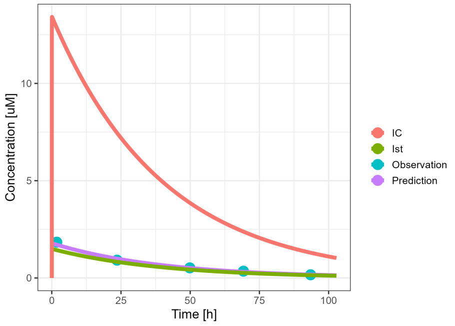

(b) Lorazepam

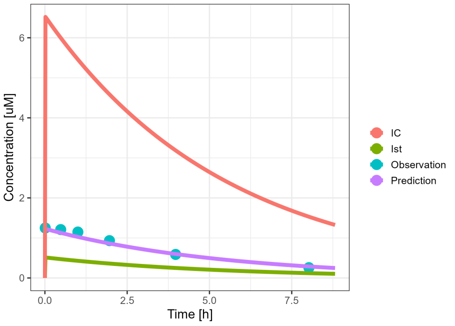

(c) Midazolam

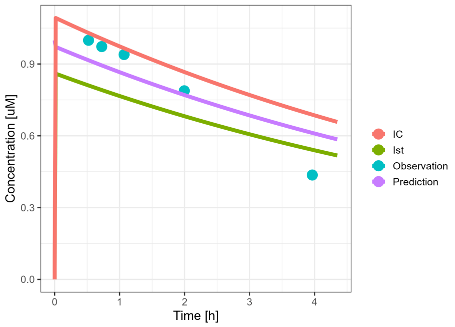

(d) Naloxone

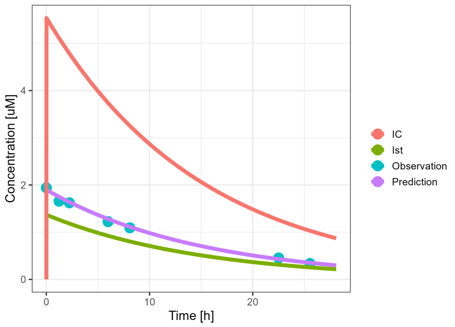

(e) Oxazepam

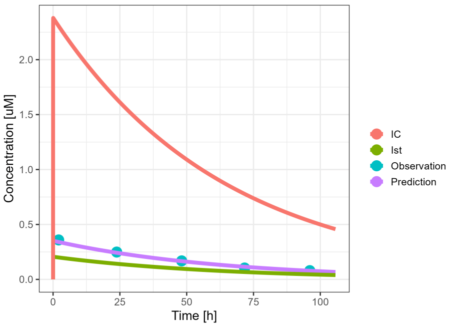

(f) Posaconazole

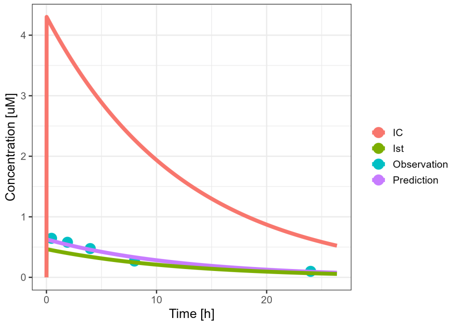

(g) Quinidine

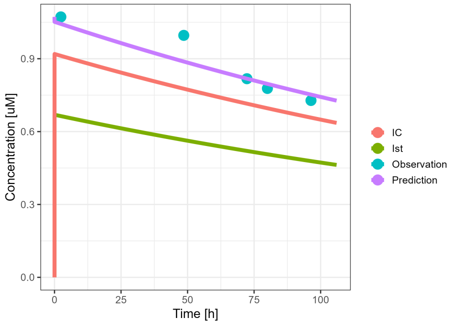

(h) Tolbutamide

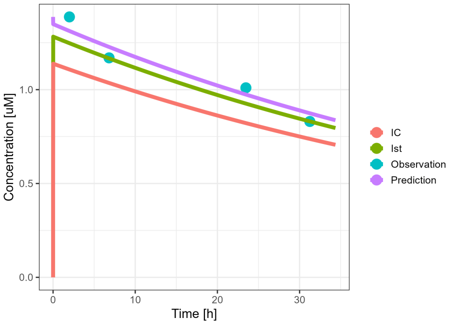

(i) Zidovudine

Fig A. Digital twin-based model simulation of on-chip kinetics after fitting parameters for 9 compounds from Docci et al [1]; IC = intracellular, Ist = interstitium.

Human PBPK Modelling of Propranolol

The Open Systems Pharmacology Suite (OSPS) (PK-Sim and MoBi) provides functionality for the full range of PBPK applications from physicochemistry-based prediction models for all relevant preclinical and clinical species to elaborated simulations for drug-drug interactions and special populations. The suite comprises functionalities for parameter identification, model qualification, and automated reporting as well as interfaces to R (The R Project for Statistical Computing, www.R-project.org) and PK-Sim release 11.3 (http://www.open-systems-pharmacology.org/). The methodological framework and the systematic presentation of findings adhere to the criteria stipulated by the EMA, FDA, and OECD in their guidelines for PBPK M&S reporting [6, 7, 8].

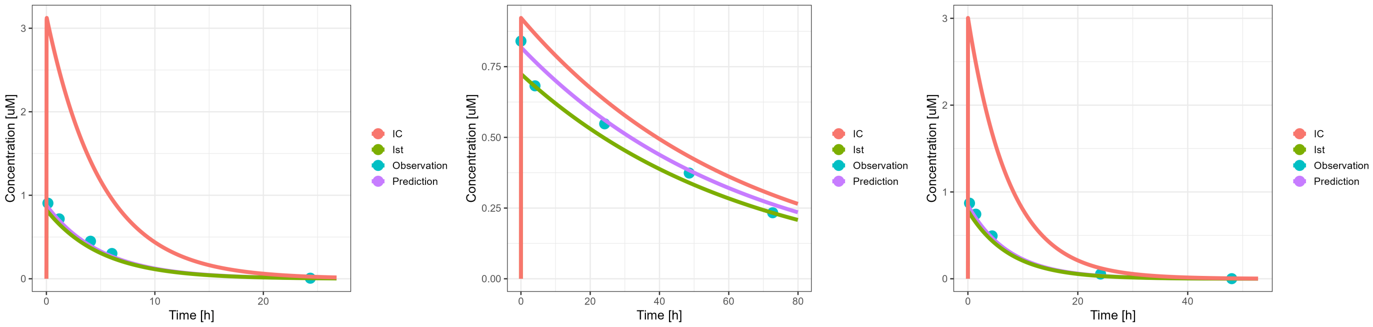

(a) Phenacetin **(b)** Prednisolone **(c)** Propranolol

Fig B. Digital twin-based model simulation of on-chip kinetics after fitting parameters for 3 compounds from Tsamandourous et al. [2]; IC = intracellular, Ist = interstitium.

In a comprehensive PBPK model (illustrated in Fig H), there is a detailed depiction of key organs involved in the drug's absorption, distribution, metabolism, and excretion [9]. These organs typically include the heart, lungs, brain, stomach, spleen, pancreas, intestines, liver, kidneys, gonads, thymus, adipose tissue, muscles, bones, and skin. The model takes into account each organ's physiological/pharmacological role, volume [10], composition, surface area, and protein expression levels. These tissues are interconnected through arterial and venous blood compartments, each characterized by unique attributes such as blood-flow rate, lymph-flow rate, volume, vascular, interstitial and intracellular fractions, tissue-partition coefficients, and permeability.

Thus, PBPK models provide an intricate structural representation of physiological mechanisms, with most parameters derived from existing knowledge bases or from rigorously validated equations. This allows for a clear differentiation between pre-established organism parameters and drug-specific parameters. Despite the complexity of PBPK models, often involving hundreds of ordinary differential equations, the number of independent parameters for a new drug is typically limited (generally less than five per drug), thanks to the extensive integration of existing physiological data. Similar to distribution models, these drug-specific parameters are typically consistent across various species or administration methods.

Drug characteristics like lipophilicity, solubility, and molecular weight are entirely independent of the organism's physiology. Conversely, drug-biological properties, such as unbound drug fraction in plasma or the tissue-plasma partition coefficient, are unique to the drug but also influenced by the drug's interaction with the biological system. By integrating these drug characteristics with the organism's anatomical and physiological traits, it becomes feasible to estimate parameters for passive processes that govern drug distribution in the body, such as membrane permeation. Additionally, data on the administration protocol and formulation attributes are crucial for configuring a PBPK simulation. The model can also incorporate temporal factors like gallbladder emptying or meal consumption, allowing for the assessment of their effects on drug pharmacokinetics (PK).

Properties for propranolol including absorption after oral administration, clearance, and human plasma PK were taken from literature [11, 12, 13].

Data used in this work

The clinical data digitised from sources are summarised in Table C.

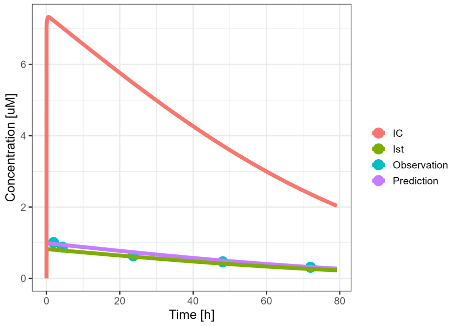

(a) Dextromethorphan

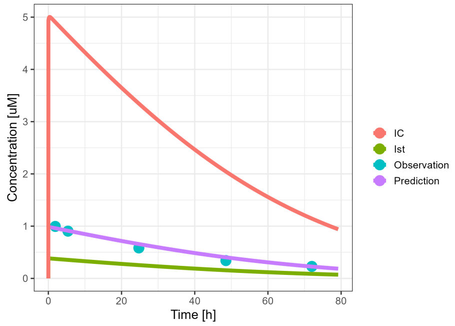

(b) Diclofenac

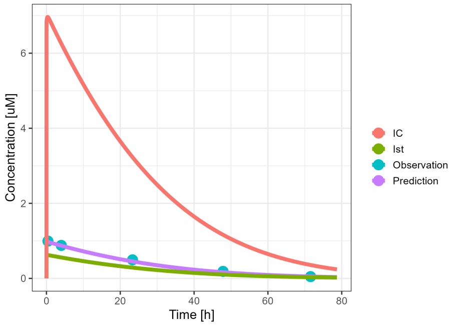

(c) Midazolam

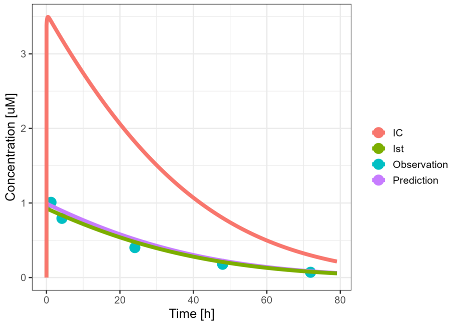

(d) Propranolol

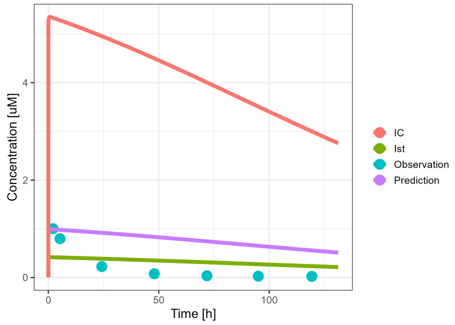

(e) Raloxifene

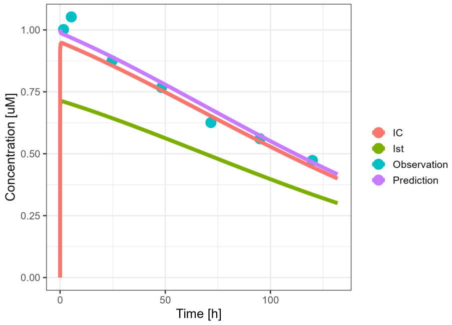

(f) Tolbutamide

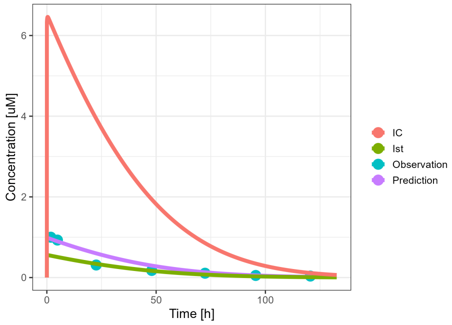

(g) Verapamil

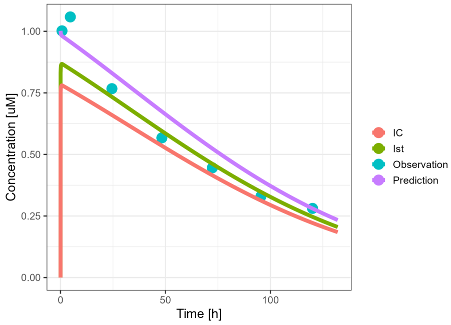

(h) Zidovudine

Fig C. Digital twin-based model simulation of on-chip kinetics after fitting parameters for 8 compounds from Rajan et al. [3]; IC = intracellular, Ist = interstitium.

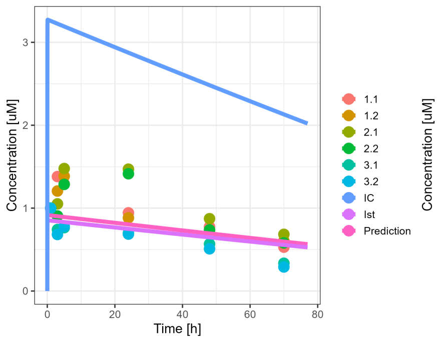

(a) (S)-warfarin [4]

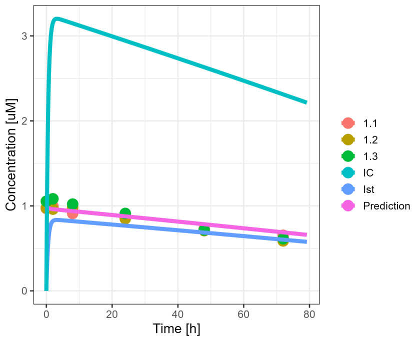

(b) (S)-warfarin [5]

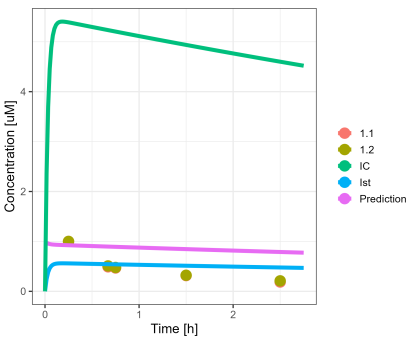

(c) Carvedilol [4]

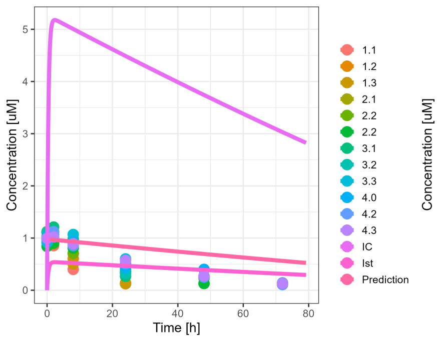

(d) Carvedilol [5]

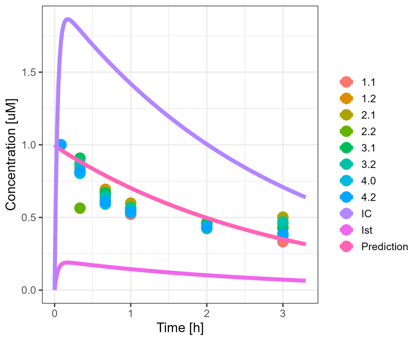

(e) Clozapine [4]

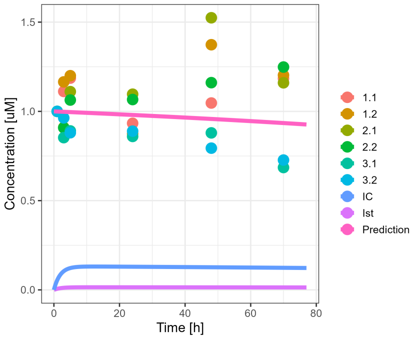

(f) Disopyramide [4]

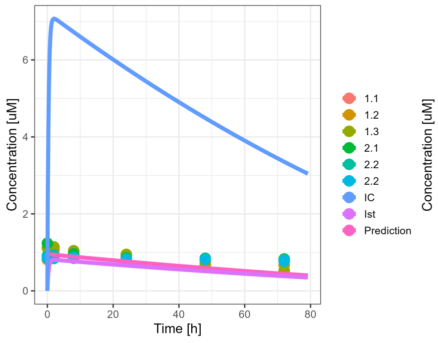

(g) Disopyramide [5]

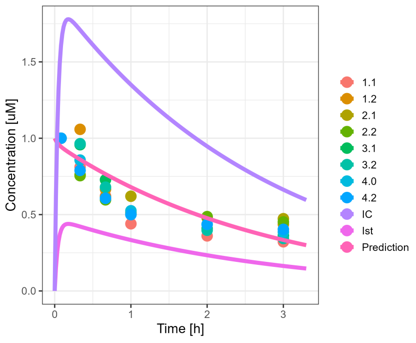

(h) Imipramine [4]

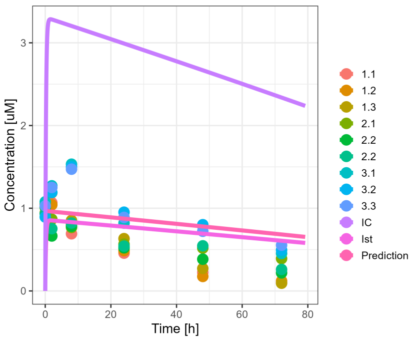

(i) Imipramine [5]

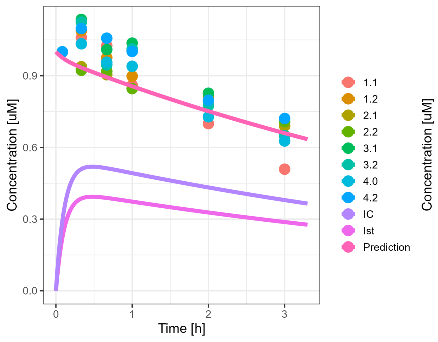

(j) Irbesartan [4]

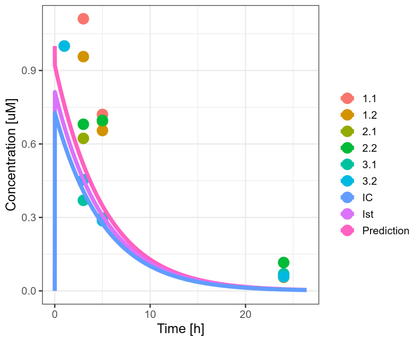

(k) Ketoprofen [4]

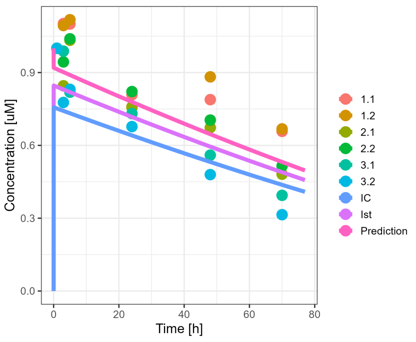

(l) Paracetamol [4]

Fig D. Digital twin-based model simulation of on-chip kinetics after fitting parameters for 3D spheroids (Kanebratt et al. [5], Bonn et al. [4]); IC = intracellular, Ist = interstitium. Numbers indicate individual biological replicates.

Names	Lipophilicty	Molecular weight $[g/mol]$	pKa	Species	Unbound fraction plasma	Unbound fraction blood	Blood-to-plasma ratio (Rbp)	Unbound fraction media
Diclofenac	4.36	318.14	4.00	Acid	0.01	0.018	0.55	0.045
Lorazepam	3.10	321.16	10.61	Neutral	0.11	0.11	1.00	0.80
Midazolam	4.32	325.77	-	Neutral	0.03	0.06	0.55	0.10
Naloxone	1.30	327.38	10.07	Neutral	0.56	0.46	1.22	0.87
Oxazepam	2.45	286.72	10.61	Neutral	0.05	0.04	1.10	0.60
Posaconazole	4.57	700.79	-	Neutral	0.02	0.02	1.00	0.37
Quinidine	3.17	324.42	13.89	Base	0.13	0.15	0.87	0.63
Tolbutamide	1.78	270.35	4.33	Acid	0.02	0.03	0.75	0.45
Zidovudine	-0.20	267.25	9.96	Neutral	0.74	0.75	0.99	0.98
Propranolol	2.58	259.35	14.9	Base	-	0.14	-	0.95
Prednisolone	1.56	360.45	12.59	Neutral	-	0.10	-	0.87
Phenacetin	2.04	179.22	-	Neutral	-	0.6	-	1.00
Imipramine	2.30	280.40	9.20	Base	0.03	0.13	0.23	0.59
Clozapine	3.627	326.80	3.70	Base	-	0.07	-	0.35
Paracetamol	0.29	151.16	9.50	Neutral	-	0.04	-	0.93
Irbesartan	1.30	428.50	7.40	Zwitter	-	0.72	-	0.52
Ketoprofen	-0.15	254.80	4.45	Acid	-	0.01	-	0.87
Disopyramide	3.10	339.50	10.42	Base	-	0.16	-	0.83
(S)-Warfarin	2.74	308.30	5.00	Neutral	-	0.01	-	0.95
Carvedilol	3.10	406.50	8.10	Base	-	0.03	-	0.43
Imipramine	2.30	280.40	9.20	Base	0.03	0.13	0.23	0.92
Disopyramide	3.10	339.50	10.42	Base	-	0.16	-	0.98
(S)-Warfarin	2.74	308.30	5.00	Neutral	-	0.01	-	0.95
Carvedilol	3.10	406.50	8.10	Base	-	0.03	-	0.43
Midazolam	4.32	325.77	-	Neutral	0.03	0.10	0.55	0.49
Dextromethorphan	3.50	271.00	9.85	Base	0.46	0.38	1.20	0.79
Diclofenac	4.36	318.14	4.00	Acid	0.01	0.01	0.55	0.04
Propranolol	2.58	259.35	14.9	Base	-	0.14	-	0.98
Raloxifene	4.60	473.00	9.00	Base	0.02	0.02	0.78	0.09
Tolbutamide	1.78	270.35	4.33	Acid	0.02	0.05	0.75	0.60
Verapamil	4.00	454.00	9.68	Base	0.19	0.28	0.67	0.35
Zidovudine	-0.20	267.25	9.96	Neutral	0.74	0.75	0.99	0.88

Table A. Drug-specific information of 32 compounds involved in the study.

**Study ID**	**Compound**	**Time**	**Time unit**	**Measurement [uM]**
Docci	Diclofenac	0.628205	h	1.021328
Docci	Diclofenac	1.979048	h	0.846287
Docci	Diclofenac	3.999128	h	0.789714
Docci	Diclofenac	7.978394	h	0.610732
Docci	Diclofenac	23.08017	h	0.368592
Docci	Lorazepam	1.793679	h	1.840524
Docci	Lorazepam	23.57192	h	0.916316
Docci	Lorazepam	49.84993	h	0.5198
Docci	Lorazepam	69.22937	h	0.349798
Docci	Lorazepam	93.48811	h	0.164888
Docci	Midazolam	0.01	h	1.248483
Docci	Midazolam	0.479718	h	1.208929

Docci	Midazolam	1.001764	h	1.147627
Docci	Midazolam	1.961199	h	0.933012
Docci	Midazolam	3.971781	h	0.587985
Docci	Midazolam	8.021164	h	0.257835
Docci	Naloxone	0.521446	h	0.999052
Docci	Naloxone	0.724757	h	0.972677
Docci	Naloxone	1.067925	h	0.939437
Docci	Naloxone	1.995312	h	0.787917
Docci	Naloxone	3.964209	h	0.436066
Docci	Oxazepam	0	h	1.941518
Docci	Oxazepam	1.232056	h	1.656614
Docci	Oxazepam	2.234957	h	1.6225
Docci	Oxazepam	5.980472	h	1.223613
Docci	Oxazepam	8.093773	h	1.092755
Docci	Oxazepam	22.51418	h	0.454003
Docci	Oxazepam	25.53957	h	0.337281
Tsamandouras	Phenacetin	0.13957	h	0.904883
Tsamandouras	Phenacetin	1.181169	h	0.717167
Tsamandouras	Phenacetin	4.064538	h	0.449706
Tsamandouras	Phenacetin	6.039438	h	0.302751
Tsamandouras	Phenacetin	24.33066	h	0.006771
Docci	Posaconazole	2.09616	h	0.359127
Docci	Posaconazole	23.80523	h	0.248864
Docci	Posaconazole	48.12191	h	0.168591
Docci	Posaconazole	71.67429	h	0.103664
Docci	Posaconazole	96.06437	h	0.079811
Tsamandouras	Prednisolone	0	h	0.840439
Tsamandouras	Prednisolone	4.060265	h	0.682204
Tsamandouras	Prednisolone	24.17242	h	0.547868
Tsamandouras	Prednisolone	48.5903	h	0.373758
Tsamandouras	Prednisolone	72.68576	h	0.233441
Tsamandouras	Propranolol	0.293087	h	0.870763
Tsamandouras	Propranolol	1.435981	h	0.744242
Tsamandouras	Propranolol	4.407433	h	0.493948
Tsamandouras	Propranolol	24.14301	h	0.054087
Tsamandouras	Propranolol	48.00981	h	0.001266
Docci	Quinidine	0.461758	h	0.644912
Docci	Quinidine	1.906867	h	0.579317
Docci	Quinidine	3.976749	h	0.475386
Docci	Quinidine	7.993031	h	0.269526
Docci	Quinidine	23.99483	h	0.099183
Docci	Tolbutamide	2.383687	h	1.072141
Docci	Tolbutamide	48.57379	h	0.996138
Docci	Tolbutamide	72.307	h	0.817721
Docci	Tolbutamide	80.00128	h	0.7776
Docci	Tolbutamide	96.37652	h	0.72809
Docci	Zidovudine	1.990311	h	1.387556
Docci	Zidovudine	6.813492	h	1.169339
Docci	Zidovudine	23.46676	h	1.009825
Docci	Zidovudine	31.24859	h	0.830464
Bonn	Carvedilol	15	min	1
Bonn	Carvedilol	40.2	min	0.49

Bonn	Carvedilol	45	min	0.47
Bonn	Carvedilol	90	min	0.32
Bonn	Carvedilol	150	min	0.19
Bonn	Carvedilol	15	min	1
Bonn	Carvedilol	40.2	min	0.51
Bonn	Carvedilol	45	min	0.48
Bonn	Carvedilol	90	min	0.32
Bonn	Carvedilol	150	min	0.21
Hultman	Imipramine	5	min	1
Hultman	Imipramine	20	min	0.8114
Hultman	Imipramine	40	min	0.6221
Hultman	Imipramine	60	min	0.4398
Hultman	Imipramine	120	min	0.3606
Hultman	Imipramine	180	min	0.322
Hultman	Imipramine	5	min	1
Hultman	Imipramine	20	min	1.0583
Hultman	Imipramine	40	min	0.6659
Hultman	Imipramine	60	min	0.5161
Hultman	Imipramine	120	min	0.4792
Hultman	Imipramine	180	min	0.4361
Hultman	Imipramine	5	min	1
Hultman	Imipramine	20	min	0.761
Hultman	Imipramine	40	min	0.6131
Hultman	Imipramine	60	min	0.6202
Hultman	Imipramine	120	min	0.4821
Hultman	Imipramine	180	min	0.4731
Hultman	Imipramine	5	min	1
Hultman	Imipramine	20	min	0.755
Hultman	Imipramine	40	min	0.5967
Hultman	Imipramine	60	min	0.5086
Hultman	Imipramine	120	min	0.4868
Hultman	Imipramine	180	min	0.4517
Hultman	Imipramine	5	min	1
Hultman	Imipramine	20	min	0.9576
Hultman	Imipramine	40	min	0.7282
Hultman	Imipramine	60	min	0.5022
Hultman	Imipramine	120	min	0.4087
Hultman	Imipramine	180	min	0.3627
Hultman	Imipramine	5	min	1
Hultman	Imipramine	20	min	0.9642
Hultman	Imipramine	40	min	0.6796
Hultman	Imipramine	60	min	0.5013
Hultman	Imipramine	120	min	0.3976
Hultman	Imipramine	180	min	0.3437
Hultman	Imipramine	5	min	1
Hultman	Imipramine	20	min	0.8569
Hultman	Imipramine	40	min	0.6061
Hultman	Imipramine	60	min	0.5251
Hultman	Imipramine	120	min	0.4353
Hultman	Imipramine	180	min	0.3858
Hultman	Imipramine	5	min	1
Hultman	Imipramine	20	min	0.7894

Hultman	Imipramine	40	min	0.6072
Hultman	Imipramine	60	min	0.4998
Hultman	Imipramine	120	min	0.4368
Hultman	Imipramine	180	min	0.4035
Hultman	Clozapine	5	min	1
Hultman	Clozapine	20	min	0.8159
Hultman	Clozapine	40	min	0.6172
Hultman	Clozapine	60	min	0.5214
Hultman	Clozapine	120	min	0.4251
Hultman	Clozapine	180	min	0.333
Hultman	Clozapine	5	min	1
Hultman	Clozapine	20	min	0.8404
Hultman	Clozapine	40	min	0.6936
Hultman	Clozapine	60	min	0.5541
Hultman	Clozapine	120	min	0.4339
Hultman	Clozapine	180	min	0.4291
Hultman	Clozapine	5	min	1
Hultman	Clozapine	20	min	0.8184
Hultman	Clozapine	40	min	0.6824
Hultman	Clozapine	60	min	0.5986
Hultman	Clozapine	120	min	0.4591
Hultman	Clozapine	180	min	0.5022
Hultman	Clozapine	5	min	1
Hultman	Clozapine	20	min	0.5641
Hultman	Clozapine	40	min	0.6443
Hultman	Clozapine	60	min	0.5512
Hultman	Clozapine	120	min	0.4326
Hultman	Clozapine	180	min	0.4569
Hultman	Clozapine	5	min	1
Hultman	Clozapine	20	min	0.9079
Hultman	Clozapine	40	min	0.6688
Hultman	Clozapine	60	min	0.5677
Hultman	Clozapine	120	min	0.4639
Hultman	Clozapine	180	min	0.4257
Hultman	Clozapine	5	min	1
Hultman	Clozapine	20	min	0.8682
Hultman	Clozapine	40	min	0.6437
Hultman	Clozapine	60	min	0.5613
Hultman	Clozapine	120	min	0.4241
Hultman	Clozapine	180	min	0.469
Hultman	Clozapine	5	min	1
Hultman	Clozapine	20	min	0.8036
Hultman	Clozapine	40	min	0.592
Hultman	Clozapine	60	min	0.5326
Hultman	Clozapine	120	min	0.4313
Hultman	Clozapine	180	min	0.3756
Hultman	Clozapine	5	min	1
Hultman	Clozapine	20	min	0.8199
Hultman	Clozapine	40	min	0.6096
Hultman	Clozapine	60	min	0.5371
Hultman	Clozapine	120	min	0.4364
Hultman	Clozapine	180	min	0.3859

Hultman	Paracetamol	60	min	1
Hultman	Paracetamol	180	min	1.1018
Hultman	Paracetamol	300	min	1.0998
Hultman	Paracetamol	1440	min	0.8085
Hultman	Paracetamol	2880	min	0.7884
Hultman	Paracetamol	4200	min	0.6577
Hultman	Paracetamol	60	min	1
Hultman	Paracetamol	180	min	1.0937
Hultman	Paracetamol	300	min	1.1177
Hultman	Paracetamol	1440	min	0.7592
Hultman	Paracetamol	2880	min	0.8823
Hultman	Paracetamol	4200	min	0.6679
Hultman	Paracetamol	60	min	1
Hultman	Paracetamol	180	min	0.8455
Hultman	Paracetamol	300	min	1.033
Hultman	Paracetamol	1440	min	0.7561
Hultman	Paracetamol	2880	min	0.6731
Hultman	Paracetamol	4200	min	0.4812
Hultman	Paracetamol	60	min	1
Hultman	Paracetamol	180	min	0.9438
Hultman	Paracetamol	300	min	1.039
Hultman	Paracetamol	1440	min	0.8216
Hultman	Paracetamol	2880	min	0.7042
Hultman	Paracetamol	4200	min	0.5152
Hultman	Paracetamol	60	min	1
Hultman	Paracetamol	180	min	0.9882
Hultman	Paracetamol	300	min	0.8191
Hultman	Paracetamol	1440	min	0.731
Hultman	Paracetamol	2880	min	0.5598
Hultman	Paracetamol	4200	min	0.3942
Hultman	Paracetamol	60	min	1
Hultman	Paracetamol	180	min	0.7768
Hultman	Paracetamol	300	min	0.8309
Hultman	Paracetamol	1440	min	0.6775
Hultman	Paracetamol	2880	min	0.4796
Hultman	Paracetamol	4200	min	0.3145
Hultman	Irbesartan	5	min	1
Hultman	Irbesartan	20	min	1.0598
Hultman	Irbesartan	40	min	1.0257
Hultman	Irbesartan	60	min	0.8607
Hultman	Irbesartan	120	min	0.6997
Hultman	Irbesartan	180	min	0.5088
Hultman	Irbesartan	5	min	1
Hultman	Irbesartan	20	min	1.0882
Hultman	Irbesartan	40	min	0.9757
Hultman	Irbesartan	60	min	0.8982
Hultman	Irbesartan	120	min	0.7932
Hultman	Irbesartan	180	min	0.69
Hultman	Irbesartan	5	min	1
Hultman	Irbesartan	20	min	0.9378
Hultman	Irbesartan	40	min	0.9043
Hultman	Irbesartan	60	min	1.0063

Hultman	Irbesartan	120	min	0.8129
Hultman	Irbesartan	180	min	0.6898
Hultman	Irbesartan	5	min	1
Hultman	Irbesartan	20	min	0.923
Hultman	Irbesartan	40	min	0.9184
Hultman	Irbesartan	60	min	0.8461
Hultman	Irbesartan	120	min	0.8265
Hultman	Irbesartan	180	min	0.7074
Hultman	Irbesartan	5	min	1
Hultman	Irbesartan	20	min	1.1356
Hultman	Irbesartan	40	min	1.0094
Hultman	Irbesartan	60	min	1.0366
Hultman	Irbesartan	120	min	0.8268
Hultman	Irbesartan	180	min	0.6495
Hultman	Irbesartan	5	min	1
Hultman	Irbesartan	20	min	1.1247
Hultman	Irbesartan	40	min	0.9454
Hultman	Irbesartan	60	min	1.0124
Hultman	Irbesartan	120	min	0.7746
Hultman	Irbesartan	180	min	0.6488
Hultman	Irbesartan	5	min	1
Hultman	Irbesartan	20	min	1.0337
Hultman	Irbesartan	40	min	0.9567
Hultman	Irbesartan	60	min	0.9397
Hultman	Irbesartan	120	min	0.7283
Hultman	Irbesartan	180	min	0.627
Hultman	Irbesartan	5	min	1
Hultman	Irbesartan	20	min	1.0975
Hultman	Irbesartan	40	min	1.0572
Hultman	Irbesartan	60	min	1.0019
Hultman	Irbesartan	120	min	0.7975
Hultman	Irbesartan	180	min	0.7211
Hultman	Ketoprofen	60	min	1
Hultman	Ketoprofen	180	min	1.1113
Hultman	Ketoprofen	300	min	0.7207
Hultman	Ketoprofen	1440	min	0.0678
Hultman	Ketoprofen	60	min	1
Hultman	Ketoprofen	180	min	0.9567
Hultman	Ketoprofen	300	min	0.6549
Hultman	Ketoprofen	1440	min	0.0554
Hultman	Ketoprofen	60	min	1
Hultman	Ketoprofen	180	min	0.6228
Hultman	Ketoprofen	300	min	0.6929
Hultman	Ketoprofen	1440	min	0.1156
Hultman	Ketoprofen	60	min	1
Hultman	Ketoprofen	180	min	0.6805
Hultman	Ketoprofen	300	min	0.6964
Hultman	Ketoprofen	1440	min	0.1157
Hultman	Ketoprofen	60	min	1
Hultman	Ketoprofen	180	min	0.3698
Hultman	Ketoprofen	300	min	0.2971
Hultman	Ketoprofen	1440	min	0.0697

Hultman	Ketoprofen	60	min	1
Hultman	Ketoprofen	180	min	0.4539
Hultman	Ketoprofen	300	min	0.2865
Hultman	Ketoprofen	1440	min	0.0578
Hultman	Disopyramide	60	min	1
Hultman	Disopyramide	180	min	1.1116
Hultman	Disopyramide	300	min	1.1863
Hultman	Disopyramide	1440	min	0.9333
Hultman	Disopyramide	2880	min	1.0469
Hultman	Disopyramide	4200	min	1.1844
Hultman	Disopyramide	60	min	1
Hultman	Disopyramide	180	min	1.1651
Hultman	Disopyramide	300	min	1.1999
Hultman	Disopyramide	1440	min	0.875
Hultman	Disopyramide	2880	min	1.3734
Hultman	Disopyramide	4200	min	1.2029
Hultman	Disopyramide	60	min	1
Hultman	Disopyramide	180	min	0.9101
Hultman	Disopyramide	300	min	1.1106
Hultman	Disopyramide	1440	min	1.0963
Hultman	Disopyramide	2880	min	1.5244
Hultman	Disopyramide	4200	min	1.16
Hultman	Disopyramide	60	min	1
Hultman	Disopyramide	180	min	0.908
Hultman	Disopyramide	300	min	1.0635
Hultman	Disopyramide	1440	min	1.0663
Hultman	Disopyramide	2880	min	1.1609
Hultman	Disopyramide	4200	min	1.2484
Hultman	Disopyramide	60	min	1
Hultman	Disopyramide	180	min	0.8529
Hultman	Disopyramide	300	min	0.8905
Hultman	Disopyramide	1440	min	0.8596
Hultman	Disopyramide	2880	min	0.8795
Hultman	Disopyramide	4200	min	0.6855
Hultman	Disopyramide	60	min	1
Hultman	Disopyramide	180	min	0.9634
Hultman	Disopyramide	300	min	0.8803
Hultman	Disopyramide	1440	min	0.8891
Hultman	Disopyramide	2880	min	0.794
Hultman	Disopyramide	4200	min	0.7273
Hultman	(S)-Warfarin	60	min	1
Hultman	(S)-Warfarin	180	min	1.3807
Hultman	(S)-Warfarin	300	min	1.2928
Hultman	(S)-Warfarin	1440	min	0.9432
Hultman	(S)-Warfarin	2880	min	0.7753
Hultman	(S)-Warfarin	4200	min	0.5294
Hultman	(S)-Warfarin	60	min	1
Hultman	(S)-Warfarin	180	min	1.2065
Hultman	(S)-Warfarin	300	min	1.3853
Hultman	(S)-Warfarin	1440	min	0.8829
Hultman	(S)-Warfarin	2880	min	0.6987
Hultman	(S)-Warfarin	4200	min	0.6049

Hultman	(S)-Warfarin	60	min	1
Hultman	(S)-Warfarin	180	min	1.0515
Hultman	(S)-Warfarin	300	min	1.4776
Hultman	(S)-Warfarin	1440	min	1.4702
Hultman	(S)-Warfarin	2880	min	0.8724
Hultman	(S)-Warfarin	4200	min	0.684
Hultman	(S)-Warfarin	60	min	1
Hultman	(S)-Warfarin	180	min	0.9055
Hultman	(S)-Warfarin	300	min	1.2863
Hultman	(S)-Warfarin	1440	min	1.4148
Hultman	(S)-Warfarin	2880	min	0.7379
Hultman	(S)-Warfarin	4200	min	0.5801
Hultman	(S)-Warfarin	60	min	1
Hultman	(S)-Warfarin	180	min	0.7413
Hultman	(S)-Warfarin	300	min	0.7643
Hultman	(S)-Warfarin	1440	min	0.6989
Hultman	(S)-Warfarin	2880	min	0.5672
Hultman	(S)-Warfarin	4200	min	0.3351
Hultman	(S)-Warfarin	60	min	1
Hultman	(S)-Warfarin	180	min	0.6822
Hultman	(S)-Warfarin	300	min	0.7794
Hultman	(S)-Warfarin	1440	min	0.6867
Hultman	(S)-Warfarin	2880	min	0.5091
Hultman	(S)-Warfarin	4200	min	0.2903
Kanebratt	Carvedilol	0	min	0.980032
Kanebratt	Carvedilol	120	min	0.853704
Kanebratt	Carvedilol	480	min	0.399648
Kanebratt	Carvedilol	1440	min	0.126797
Kanebratt	Carvedilol	2880	min	
Kanebratt	Carvedilol	4320	min	
Kanebratt	Carvedilol	0	min	0.909218
Kanebratt	Carvedilol	120	min	0.98593
Kanebratt	Carvedilol	480	min	0.605821
Kanebratt	Carvedilol	1440	min	0.138046
Kanebratt	Carvedilol	2880	min	
Kanebratt	Carvedilol	4320	min	
Kanebratt	Carvedilol	0	min	1.110522
Kanebratt	Carvedilol	120	min	0.992855
Kanebratt	Carvedilol	480	min	0.504509
Kanebratt	Carvedilol	1440	min	0.123914
Kanebratt	Carvedilol	2880	min	
Kanebratt	Carvedilol	4320	min	
Kanebratt	Carvedilol	0	min	1.036479
Kanebratt	Carvedilol	120	min	1.03131
Kanebratt	Carvedilol	480	min	0.706685
Kanebratt	Carvedilol	1440	min	0.388225
Kanebratt	Carvedilol	2880	min	0.128713
Kanebratt	Carvedilol	4320	min	
Kanebratt	Carvedilol	0	min	1.123928
Kanebratt	Carvedilol	120	min	0.891214
Kanebratt	Carvedilol	480	min	0.704568
Kanebratt	Carvedilol	1440	min	0.334817

Kanebratt	Carvedilol	2880	min	0.229886
Kanebratt	Carvedilol	4320	min	
Kanebratt	Carvedilol	0	min	0.840154
Kanebratt	Carvedilol	120	min	0.909218
Kanebratt	Carvedilol	480	min	0.810446
Kanebratt	Carvedilol	1440	min	0.350929
Kanebratt	Carvedilol	2880	min	0.129358
Kanebratt	Carvedilol	4320	min	
Kanebratt	Carvedilol	0	min	0.971251
Kanebratt	Carvedilol	120	min	1.210253
Kanebratt	Carvedilol	480	min	0.981012
Kanebratt	Carvedilol	1440	min	0.26709
Kanebratt	Carvedilol	2880	min	
Kanebratt	Carvedilol	4320	min	
Kanebratt	Carvedilol	0	min	1.112745
Kanebratt	Carvedilol	120	min	1.122805
Kanebratt	Carvedilol	480	min	0.902876
Kanebratt	Carvedilol	1440	min	0.322011
Kanebratt	Carvedilol	2880	min	
Kanebratt	Carvedilol	4320	min	
Kanebratt	Carvedilol	0	min	0.915605
Kanebratt	Carvedilol	120	min	1.066977
Kanebratt	Carvedilol	480	min	1.068045
Kanebratt	Carvedilol	1440	min	0.600393
Kanebratt	Carvedilol	2880	min	
Kanebratt	Carvedilol	4320	min	
Kanebratt	Carvedilol	0	min	1.012912
Kanebratt	Carvedilol	120	min	1.110522
Kanebratt	Carvedilol	480	min	0.927586
Kanebratt	Carvedilol	1440	min	0.428626
Kanebratt	Carvedilol	2880	min	0.400048
Kanebratt	Carvedilol	4320	min	0.140553
Kanebratt	Carvedilol	0	min	0.989881
Kanebratt	Carvedilol	120	min	1.122805
Kanebratt	Carvedilol	480	min	0.856269
Kanebratt	Carvedilol	1440	min	0.508053
Kanebratt	Carvedilol	2880	min	0.252797
Kanebratt	Carvedilol	4320	min	0.108808
Kanebratt	Carvedilol	0	min	0.996835
Kanebratt	Carvedilol	120	min	1.021048
Kanebratt	Carvedilol	480	min	0.891214
Kanebratt	Carvedilol	1440	min	0.572255
Kanebratt	Carvedilol	2880	min	0.273031
Kanebratt	Carvedilol	4320	min	0.130789
Kanebratt	Imipramine	0	min	1.04376
Kanebratt	Imipramine	120	min	1.066977
Kanebratt	Imipramine	480	min	0.691308
Kanebratt	Imipramine	1440	min	0.457412
Kanebratt	Imipramine	2880	min	0.22421
Kanebratt	Imipramine	4320	min	0.099543
Kanebratt	Imipramine	0	min	0.944433
Kanebratt	Imipramine	120	min	1.041675

Kanebratt	Imipramine	480	min	0.803989
Kanebratt	Imipramine	1440	min	0.487156
Kanebratt	Imipramine	2880	min	0.175491
Kanebratt	Imipramine	4320	min	0.120733
Kanebratt	Imipramine	0	min	1.012912
Kanebratt	Imipramine	120	min	1.187476
Kanebratt	Imipramine	480	min	0.851999
Kanebratt	Imipramine	1440	min	0.63244
Kanebratt	Imipramine	2880	min	0.271941
Kanebratt	Imipramine	4320	min	0.093652
Kanebratt	Imipramine	0	min	1.052144
Kanebratt	Imipramine	120	min	0.866607
Kanebratt	Imipramine	480	min	0.767843
Kanebratt	Imipramine	1440	min	0.565429
Kanebratt	Imipramine	2880	min	0.518316
Kanebratt	Imipramine	4320	min	0.391343
Kanebratt	Imipramine	0	min	1.012912
Kanebratt	Imipramine	120	min	0.664866
Kanebratt	Imipramine	480	min	0.782571
Kanebratt	Imipramine	1440	min	0.521435
Kanebratt	Imipramine	2880	min	0.383978
Kanebratt	Imipramine	4320	min	0.218893
Kanebratt	Imipramine	0	min	0.934101
Kanebratt	Imipramine	120	min	0.752639
Kanebratt	Imipramine	480	min	0.821051
Kanebratt	Imipramine	1440	min	0.541631
Kanebratt	Imipramine	2880	min	0.545436
Kanebratt	Imipramine	4320	min	0.259715
Kanebratt	Imipramine	0	min	1.077701
Kanebratt	Imipramine	120	min	1.271033
Kanebratt	Imipramine	480	min	1.532392
Kanebratt	Imipramine	1440	min	0.82023
Kanebratt	Imipramine	2880	min	0.784923
Kanebratt	Imipramine	4320	min	0.453768
Kanebratt	Imipramine	0	min	0.893892
Kanebratt	Imipramine	120	min	1.191044
Kanebratt	Imipramine	480	min	1.499047
Kanebratt	Imipramine	1440	min	0.952019
Kanebratt	Imipramine	2880	min	0.800779
Kanebratt	Imipramine	4320	min	0.494519
Kanebratt	Imipramine	0	min	1.028221
Kanebratt	Imipramine	120	min	1.250858
Kanebratt	Imipramine	480	min	1.472306
Kanebratt	Imipramine	1440	min	0.889434
Kanebratt	Imipramine	2880	min	0.721682
Kanebratt	Imipramine	4320	min	0.553126
Kanebratt	Disopyramide	120	min	1.062718
Kanebratt	Disopyramide	480	min	0.920195
Kanebratt	Disopyramide	1440	min	0.912862
Kanebratt	Disopyramide	2880	min	0.756411
Kanebratt	Disopyramide	4320	min	0.51728
Kanebratt	Disopyramide	0	min	0.812069

Kanebratt	Disopyramide	120	min	1.106088
Kanebratt	Disopyramide	480	min	1.042717
Kanebratt	Disopyramide	1440	min	0.901072
Kanebratt	Disopyramide	2880	min	0.757925
Kanebratt	Disopyramide	4320	min	0.666863
Kanebratt	Disopyramide	0	min	1.116088
Kanebratt	Disopyramide	120	min	1.142056
Kanebratt	Disopyramide	480	min	1.039594
Kanebratt	Disopyramide	1440	min	0.948219
Kanebratt	Disopyramide	2880	min	0.681016
Kanebratt	Disopyramide	4320	min	0.525098
Kanebratt	Disopyramide	0	min	1.235938
Kanebratt	Disopyramide	120	min	0.844365
Kanebratt	Disopyramide	480	min	0.960626
Kanebratt	Disopyramide	1440	min	0.860562
Kanebratt	Disopyramide	2880	min	0.84521
Kanebratt	Disopyramide	4320	min	0.828474
Kanebratt	Disopyramide	0	min	0.912862
Kanebratt	Disopyramide	120	min	0.934101
Kanebratt	Disopyramide	480	min	0.846902
Kanebratt	Disopyramide	1440	min	0.841836
Kanebratt	Disopyramide	2880	min	0.825992
Kanebratt	Disopyramide	4320	min	0.809636
Kanebratt	Disopyramide	0	min	0.850296
Kanebratt	Disopyramide	120	min	0.841836
Kanebratt	Disopyramide	480	min	0.868342
Kanebratt	Disopyramide	1440	min	0.792019
Kanebratt	Disopyramide	2880	min	0.82023
Kanebratt	Disopyramide	4320	min	0.767843
Kanebratt	(S)-Warfarin	0	min	0.97028
Kanebratt	(S)-Warfarin	120	min	0.995838
Kanebratt	(S)-Warfarin	480	min	0.911038
Kanebratt	(S)-Warfarin	1440	min	0.887657
Kanebratt	(S)-Warfarin	2880	min	0.729665
Kanebratt	(S)-Warfarin	4320	min	0.654313
Kanebratt	(S)-Warfarin	0	min	0.975144
Kanebratt	(S)-Warfarin	120	min	0.961587
Kanebratt	(S)-Warfarin	480	min	0.981012
Kanebratt	(S)-Warfarin	1440	min	0.849447
Kanebratt	(S)-Warfarin	2880	min	0.708808
Kanebratt	(S)-Warfarin	4320	min	0.587917
Kanebratt	(S)-Warfarin	0	min	1.05425
Kanebratt	(S)-Warfarin	120	min	1.083103
Kanebratt	(S)-Warfarin	480	min	1.021048
Kanebratt	(S)-Warfarin	1440	min	0.911038
Kanebratt	(S)-Warfarin	2880	min	0.715932
Kanebratt	(S)-Warfarin	4320	min	0.613748
Rajan	Midazolam	0.40201	h	0.993976
Rajan	Midazolam	4.020101	h	0.879518
Rajan	Midazolam	23.31658	h	0.493976
Rajan	Midazolam	47.8392	h	0.186747
Rajan	Midazolam	71.55779	h	0.048193

Rajan	Dextromethorphan	1.942896	h	1.00512
Rajan	Dextromethorphan	4.354703	h	0.876159
Rajan	Dextromethorphan	23.68081	h	0.632832
Rajan	Dextromethorphan	48.07975	h	0.464896
Rajan	Dextromethorphan	72.02798	h	0.311109
Rajan	Diclofenac	1.895298	h	0.994907
Rajan	Diclofenac	5.364779	h	0.904459
Rajan	Diclofenac	24.72736	h	0.583287
Rajan	Diclofenac	48.51564	h	0.341034
Rajan	Diclofenac	72.00207	h	0.229646
Rajan	Propranolol	1.241641	h	1.008705
Rajan	Propranolol	4.211634	h	0.793491
Rajan	Propranolol	24.06928	h	0.401561
Rajan	Propranolol	47.96644	h	0.178121
Rajan	Propranolol	71.89563	h	0.071002
Rajan	Raloxifene	2.075782	h	1.003443
Rajan	Raloxifene	5.267301	h	0.797817
Rajan	Raloxifene	24.20649	h	0.226786
Rajan	Raloxifene	47.9725	h	0.077503
Rajan	Raloxifene	71.80673	h	0.037835
Rajan	Raloxifene	95.03639	h	0.026814
Rajan	Raloxifene	119.3087	h	0.02426
Rajan	(S)-warfarin	1.762283	h	0.996526
Rajan	(S)-warfarin	5.20745	h	0.936853
Rajan	(S)-warfarin	24.61241	h	0.932035
Rajan	(S)-warfarin	48.11634	h	0.830653
Rajan	(S)-warfarin	72.07	h	0.797822
Rajan	(S)-warfarin	96.70722	h	0.749191
Rajan	(S)-warfarin	119.5896	h	0.707547
Rajan	(S)-warfarin	144.5376	h	0.629046
Rajan	(S)-warfarin	167.8325	h	0.629592
Rajan	(S)-warfarin	192.0721	h	0.549316
Rajan	Tolbutamide	1.637369	h	1.00167
Rajan	Tolbutamide	5.328741	h	1.052478
Rajan	Tolbutamide	24.7199	h	0.87525
Rajan	Tolbutamide	48.21525	h	0.767491
Rajan	Tolbutamide	71.70365	h	0.625776
Rajan	Tolbutamide	95.00318	h	0.560469
Rajan	Tolbutamide	119.9342	h	0.473035
Rajan	Verapamil	1.814516	h	1.000085
Rajan	Verapamil	4.83871	h	0.929395
Rajan	Verapamil	22.58065	h	0.310961
Rajan	Verapamil	47.98387	h	0.176282
Rajan	Verapamil	72.37903	h	0.107396
Rajan	Verapamil	95.56452	h	0.051922
Rajan	Verapamil	120.5645	h	0.037142
Rajan	Zidovudine	0.699675	h	1.001949
Rajan	Zidovudine	4.670238	h	1.058512
Rajan	Zidovudine	24.48861	h	0.767109
Rajan	Zidovudine	48.29386	h	0.567425
Rajan	Zidovudine	72.36738	h	0.445861
Rajan	Zidovudine	95.51556	h	0.330175

Rajan	Zidovudine	120.0866	h	0.280867

Table C. Summary of kinetic data used in this work: study ID, compound name, time of measurement and concentration values.

References

1. Docci L, Milani N, Ramp T, Romeo AA, Godoy P, Franyuti DO, et al. Exploration and application of a liver-on-a-chip device in combination with modelling and simulation for quantitative drug metabolism studies. Lab on a Chip. 2022;22(6):1187–1205. doi:10.1039/d1lc01161h.

2. Tsamandouras N, Kostrzewski T, Stokes CL, Griffith LG, Hughes DJ, Cirit M. Quantitative assessment of population variability in hepatic drug metabolism using a perfused three-dimensional human liver microphysiological system. Journal of Pharmacology and Experimental Therapeutics. 2016;360(1):95–105. doi:10.1124/jpet.116.237495.

3. Rajan SAP, Sherfey J, Ohri S, Nichols L, Smith JT, Parekh P, et al. A novel milli-fluidic liver tissue chip with continuous recirculation for predictive pharmacokinetics applications. The AAPS Journal. 2023;25(6). doi:10.1208/s12248-023-00870-x.

4. Bonn B, Svanberg P, Janefeldt A, Hultman I, Grime K. Determination of human hepatocyte intrinsic clearance for slowly metabolized compounds: comparison of a primary hepatocyte/stromal cell co-culture with plated primary hepatocytes and HepaRG. Drug Metabolism and Disposition. 2016;44(4):527–533. doi:10.1124/dmd.115.067769.

5. Kanebratt KP, Janefeldt A, Vilén L, Vildhede A, Samuelsson K, Milton L, et al. Primary human hepatocyte spheroid model as a 3D *in vitro* platform for metabolism studies. Journal of Pharmaceutical Sciences. 2021;110(1):422–431. doi:10.1016/j.xphs.2020.10.043.

6. European Medicines Agency. ICH S7A safety pharmacology studies for human pharmaceuticals; 2018. `https://www.ema.europa.eu/en/ich-s7a-safety-pharmacology-studies-human-pharmaceuticals`.

7. European Medicines Agency. Guideline on the investigation of bioequivalence; 2010. `https://www.ema.europa.eu/en/documents/scientific-guideline/guideline-investigation-bioequivalence-rev1_en.pdf`.

8. U S Food and Drug Administration. Physiologically based pharmacokinetic analyses — format and content guidance for industry.; 2019. `http://www.fda.gov/regulatory-information/search-fda-guidance-documents/physiologically-based-pharmacokinetic-analyses-format-and-content-guidance`

9. Thelen K, Coboeken K, Willmann S, Dressman JB, Lippert J. Evolution of a detailed physiological model to simulate the gastrointestinal transit and absorption process in humans, Part II: Extension to describe performance of solid dosage forms. Journal of Pharmaceutical Sciences. 2012;101(3):1267–1280. doi:10.1002/jps.22825.

10. Peters SA. Physiologically based pharmacokinetic (PBPK) modeling and simulations: principles, methods, and applications in the pharmaceutical industry. Hoboken, NJ: Wiley; 2011.

Names	CL_{obs} $[ml/min/kg]$	CL_{conv} $[ml/min/kg]$	CL_{pred} $[ml/min/kg]$	$\frac{CL_{pred}}{CL_{obs}}$	$\frac{CL_{conv}}{CL_{obs}}$	On-chip clearance $[ml/min]$	Surface area $[cm^2]$	Study
Diclofenac	6.83	3.62	6.78	0.99	0.53	2.55E-03	2.90E-03	Docci
Lorazepam	1.43	0.69	1.36	0.95	0.48	5.48E-04	1.26E-05	Docci
Midazolam	11.28	12.67	11.08	0.98	1.12	2.20E-03	8.90E-04	Docci
Naloxone	16.89	16.30	19.45	1.15	0.96	1.11E-02	9.50E-04	Docci
Oxazepam	1.30	1.41	1.30	0.99	1.08	1.05E-03	7.16E-05	Docci
Posaconazole	2.24	0.42	0.81	0.36	0.19	5.67E-04	1.34E-05	Docci
Quinidine	6.33	3.79	5.68	0.90	0.60	1.65E-03	3.60E-05	Docci
Tolbutamide	0.21	0.10	0.36	1.73	0.47	1.02E-04	1.30E-04	Docci
Zidovudine	14.58	3.27	14.18	0.97	0.22	9.90E-04	6.80E-06	Docci
Propranolol	10.80	1.55	13.06	1.21	0.14	2.75E-03	5.34E-05	Tsamandouras
Prednisolone	2.02	0.25	2.83	1.40	0.12	3.58E-04	2.60E-02	Tsamandouras
Phenacetin	19.50	9.33	13.08	0.67	0.48	1.48E-03	2.37E-02	Tsamandouras
Imipramine	13.81	2.12	17.83	1.29	0.15	3.10E-04	2.70E-01	Hultman
Clozapine	8.41	1.33	8.47	1.01	0.16	2.90E-04	2.20E-01	Hultman
Paracetamol	0.40	0.23	0.52	1.31	0.57	7.50E-06	7.20E-02	Hultman
Irbesartan	19.28	2.86	18.44	0.96	0.15	3.89E-04	1.00E-01	Hultman
Ketoprofen	1.28	0.01	2.58	2.01	0.01	4.11E-04	9.00E-01	Hultman
Disopyramide	0.90	0.49	0.75	0.83	0.55	1.00E-05	1.40E-04	Hultman
(S)-Warfarin	0.06	0.002	0.06	0.94	0.03	4.80E-05	2.47E-01	Hultman
Carvedilol	8.70	4.05	10.95	1.26	0.47	1.51E-04	1.04E-02	Bonn
Imipramine	13.81	4.35	14.49	1.05	0.32	1.00E-06	2.90E-02	Kanebratt
Disopyramide	0.90	1.53	0.81	0.90	1.70	7.90E-06	1.00E-01	Kanebratt
(S)-Warfarin	0.06	0.09	0.03	0.56	1.47	1.00E-06	5.30E-03	Kanebratt
Carvedilol	8.70	4.05	6.82	0.78	0.47	1.00E-06	2.00E-02	Kanebratt
Midazolam	5.30	2.87	4.61	0.87	0.54	4.80E-04	1.59E-05	Rajan
Dextromethorphan	18.40	2.68	18.77	1.02	0.15	4.24E-05	2.12E-03	Rajan
Diclofenac	3.50	3.02	3.38	0.96	0.86	8.00E-05	2.50E-03	Rajan
Propranolol	10.80	5.00	8.06	0.75	0.46	1.90E-04	3.80E-03	Rajan
Raloxifene	9.33	13.8	13.29	1.42	1.48	1.50E-05	2.29E-03	Rajan
Tolbutamide	0.21	0.24	0.23	1.08	1.14	1.10E-04	2.88E-03	Rajan
Verapamil	17.60	7.85	13.69	0.78	0.45	8.50E-05	1.60E-03	Rajan
Zidovudine	14.58	4.20	15.18	1.04	0.29	2.20E-04	7.80E-03	Rajan

Table B. Overview of observed, conventional, on-chip and predicted clearance values of the investigated 32 drugs across different *in vitro* systems.
CL = clearance, obs = observation, conv = conventional, pred = prediction.

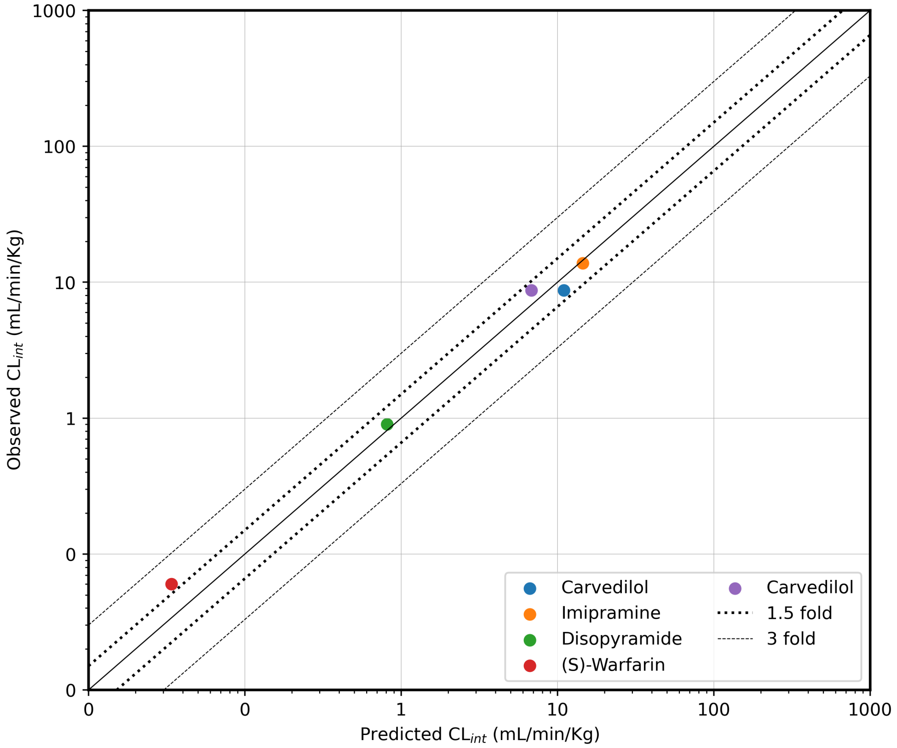

Fig E. Correlation between observed and predicted *in vivo* intrinsic clearance (CL_{int}) using three-compartment model for 4 drugs from Kanebratt et.al [5] and Carvedilol from Bonn et. al[4] (AFE = 0.879). The solid line shows the line of unity.

11. Paixão P, Gouveia LF, Morais JAG. Prediction of the in vitro permeability determined in Caco-2 cells by using artificial neural networks. European Journal of Pharmaceutical Sciences. 2010;41(1):107–117. doi:10.1016/j.ejps.2010.05.014.

12. Borgström L, Johansson CG, Larsson H, Lenander R. Pharmacokinetics of propranolol. Journal of Pharmacokinetics and Biopharmaceutics. 1981;9(4):419–429. doi:10.1007/bf01060886.

13. Zheng Y, Benet LZ, Okochi H, Chen X. pH dependent but not P-gp dependent bidirectional transport study of S-propranolol: The importance of passive diffusion. Pharmaceutical Research. 2015;doi:10.1007/s11095-015-1640-3.

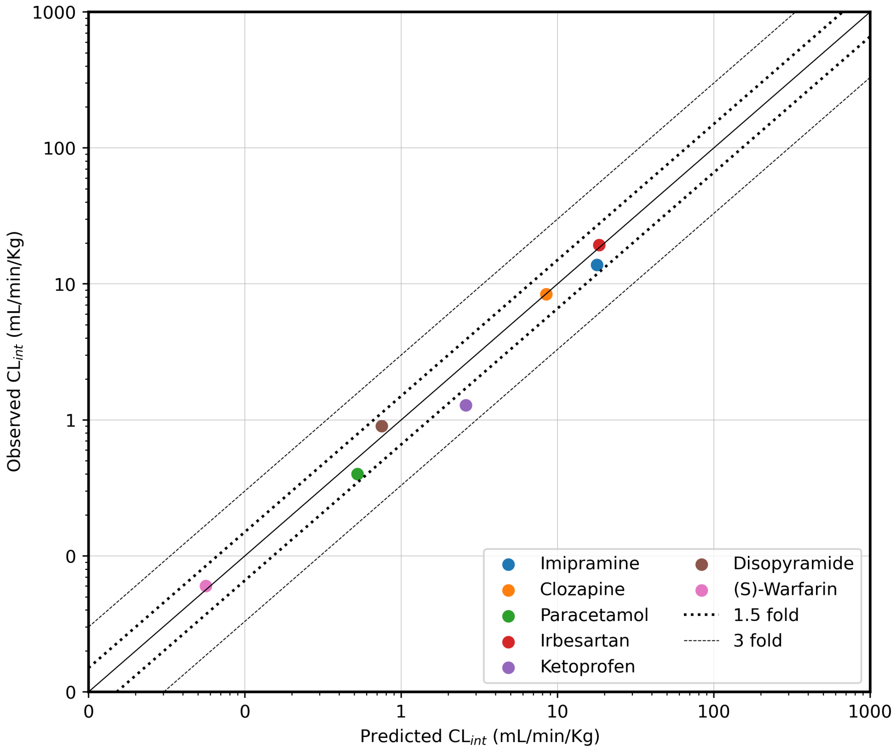

Fig F. Correlation between observed and predicted *in vivo* intrinsic clearance (CL_{int}) using three-compartment model for 7 drugs from Hultman et.al [4] (AFE= 1.143). The solid line shows the line of unity.

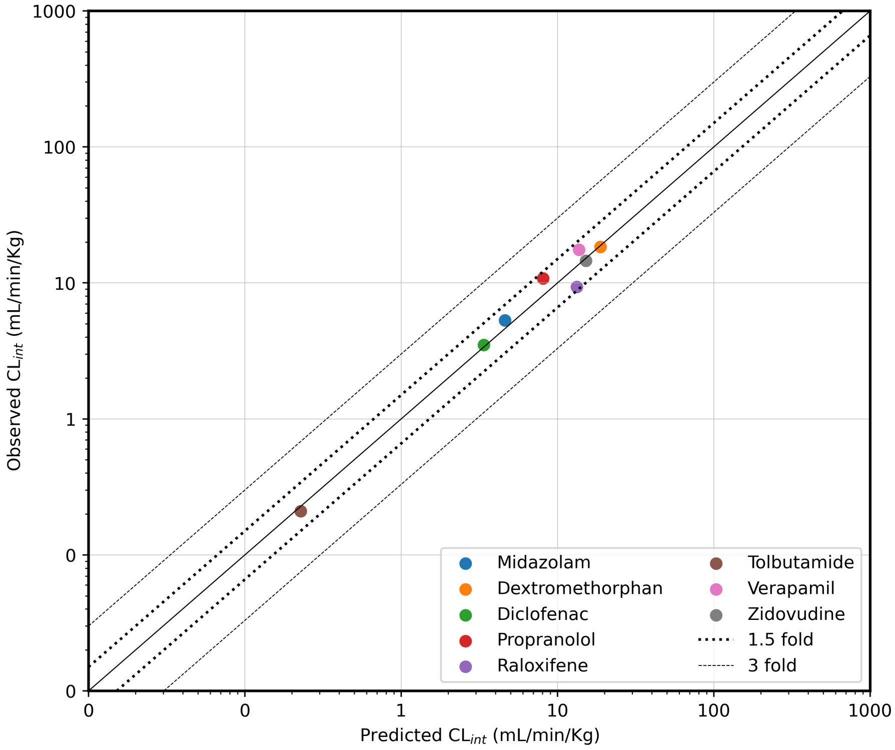

Fig G. Correlation between observed and predicted *in vivo* intrinsic clearance (CL_{int}) using three-compartment ODE liver chip for 8 drugs from Rajan et. al [3] (AFE=0.972). The solid line shows the line of unity.

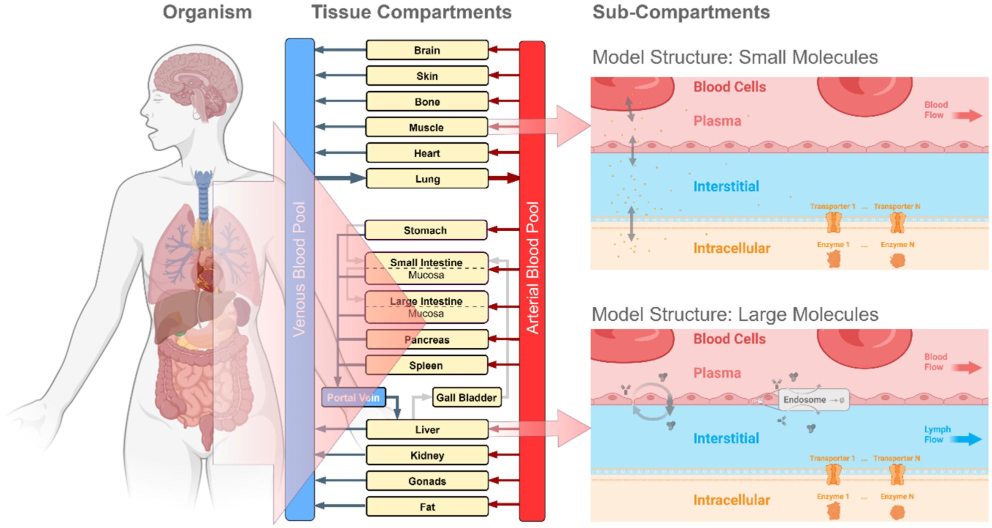

Fig H. Representation of the generic structure of a whole-body PBPK model
